# Diagnostic accuracy of symptoms for an underlying disease: a simulation study

**DOI:** 10.1038/s41598-022-14826-2

**Published:** 2022-08-15

**Authors:** Yi-Sheng Chao, Chao-Jung Wu, Yi-Chun Lai, Hui-Ting Hsu, Yen-Po Cheng, Hsing-Chien Wu, Shih-Yu Huang, Wei-Chih Chen

**Affiliations:** 1 Montreal, Canada; 2 Université du Québec à Montréal, Montreal, Canada; 3 National Yang Ming Chiao Tung University Hospital, Yilan City, Taiwan; 4 Changhua Christian Hospital, Changhua City, Taiwan; 5 Jinshan Branch, National Taiwan University Hospital, New Taipei City, Taiwan; 6 Shuang Ho Hospital, New Taipei City, Taiwan; 7 Taipei Medical University, Taipei, Taiwan; 8 Taipei Veterans General Hospital, Taipei, Taiwan; 9 National Yang Ming Chiao Tung University, Taipei, Taiwan

**Keywords:** Epidemiology, Computational models, Epidemiology, Population screening

## Abstract

Symptoms have been used to diagnose conditions such as frailty and mental illnesses. However, the diagnostic accuracy of the numbers of symptoms has not been well studied. This study aims to use equations and simulations to demonstrate how the factors that determine symptom incidence influence symptoms’ diagnostic accuracy for disease diagnosis. Assuming a disease causing symptoms and correlated with the other disease in 10,000 simulated subjects, 40 symptoms occurred based on 3 epidemiological measures: proportions diseased, baseline symptom incidence (among those not diseased), and risk ratios. Symptoms occurred with similar correlation coefficients. The sensitivities and specificities of single symptoms for disease diagnosis were exhibited as equations using the three epidemiological measures and approximated using linear regression in simulated populations. The areas under curves (AUCs) of the receiver operating characteristic (ROC) curves was the measure to determine the diagnostic accuracy of multiple symptoms, derived by using 2 to 40 symptoms for disease diagnosis. With respect to each AUC, the best set of sensitivity and specificity, whose difference with 1 in the absolute value was maximal, was chosen. The results showed sensitivities and specificities of single symptoms for disease diagnosis were fully explained with the three epidemiological measures in simulated subjects. The AUCs increased or decreased with more symptoms used for disease diagnosis, when the risk ratios were greater or less than 1, respectively. Based on the AUCs, with risk ratios were similar to 1, symptoms did not provide diagnostic values. When risk ratios were greater or less than 1, maximal or minimal AUCs usually could be reached with less than 30 symptoms. The maximal AUCs and their best sets of sensitivities and specificities could be well approximated with the three epidemiological and interaction terms, adjusted R-squared ≥ 0.69. However, the observed overall symptom correlations, overall symptom incidence, and numbers of symptoms explained a small fraction of the AUC variances, adjusted R-squared ≤ 0.03. In conclusion, the sensitivities and specificities of single symptoms for disease diagnosis can be explained fully by the at-risk incidence and the 1 minus baseline incidence, respectively. The epidemiological measures and baseline symptom correlations can explain large fractions of the variances of the maximal AUCs and the best sets of sensitivities and specificities. These findings are important for researchers who want to assess the diagnostic accuracy of composite diagnostic criteria.

## Introduction

Symptoms that are considered signs of certain diseases have been used for diagnostic purposes such as those used for the diagnosis of frailty and mental illnesses^1,2^. The diagnostic accuracy of these symptoms varies over a wide range^3^. The number of symptoms used to diagnose varies across diagnoses^4^ and even for the same diagnosis^1,5^. For example, *frailty*, a geriatric syndrome, has been defined and diagnosed with 4 to 70 symptoms, depending on the frailty models^1,6–9^. More complicated frailty models define and use more frailty symptoms for diagnosis^10^. Since various designs and model specifications coexist, it remains unclear whether there are optimal numbers of symptoms for the diagnosis of a disease, for example, frailty.

In a simplistic hypothetical case (see Table 1), a proportion of a population is affected by a disease, denoted by *d*, and a constant and random rate of incidence occurs for one symptom at a particular time point, denoted by *ir*. For those affected by the disease, the incidence rate of the symptoms increases by a risk ratio, denoted by *rr*. With respect to the individuals diseased, the proportion of those presenting the symptom was *d* × *ir* × *rr,* and the proportion of those not presenting the symptom was *d* × *(1 − ir* × *rr)*. Regarding those not diseased, the proportion of those presenting the symptom was *(1 − d)* × *ir* and the proportion of those not presenting the symptom was *(1 − d)* × *(1 − ir)*. The sensitivity^11^ of this symptom for detecting the disease equaled *(d* × *ir* × *rr)/d* = *ir* × *rr*, and the specificity was *[(1 − d)* × *(1 − ir)]/(1 − d)* = *1 − ir*. Based on these calculations, the symptom incidence (and the risk ratio) should connect to the diagnostic test accuracy of the symptoms for the disease.

**Table 1:** Diagnostic accuracy of a single symptom to diagnose the disease cause in equations.

Disease status	Disease present	Disease absent
Proportions diseased	$d$	$1-d$
Symptom present	$d \times ir \times rr$	$(1-d)\times ir$
Symptom absent	$d\times (1-ir\times rr)$	$(1-d)\times (1-ir)$
**Derived statistics if the symptom present**		
Sensitivity^11^	$\frac{d\times ir\times rr}{d}=ir\times rr$	
Specificity^11^	$\frac{\left(1-d\right)\times \left(1-ir\right)}{1-d} =1-ir$	
Positive predictive value^11^	$\frac{d\times ir\times rr}{d\times ir\times rr+\left(1-d\right)\times ir}$ = $\frac{d\times ir\times rr}{d\times ir\times rr+ir-d\times ir}$	
Negative predictive value^11^	$\frac{(1-d)\times (1-ir)}{d\times \left(1-ir\times rr\right)+(1-d)\times (1-ir)}$ = $\frac{1-d-ir+d\times ir}{1-d\times ir\times rr-ir+d\times ir}$	
Observed ratios of developing symptoms	$\frac{d\times ir\times rr+(1-d)\times ir}{d\times \left(1-ir\times rr\right)+(1-d)\times (1-ir)}$ = $\frac{d\times ir\times rr+ir-d\times ir}{1-d\times ir\times rr-ir+d\times ir}$	
**Derived statistics if the incidence reaching 1 among those diseased (**${\varvec{i}}{\varvec{r}}\times {\varvec{r}}{\varvec{r}}\,{\mathbf{=}}\,{\mathbf{1}})$		
Sensitivity^11^	$\frac{d\times ir\times rr}{d}=ir\times rr=1$	
Specificity^11^	$\frac{\left(1-d\right)\times \left(1-ir\right)}{1-d} =1-ir$	
Positive predictive value^11^	$\frac{d\times ir\times rr}{d\times ir\times rr+\left(1-d\right)\times ir}$ = $\frac{d\times ir\times rr}{d\times ir\times rr+ir-d\times ir}$ = $\frac{d}{d+ir-d\times ir}$	
Negative predictive value^11^	$\frac{(1-d)\times (1-ir)}{d\times \left(1-ir\times rr\right)+(1-d)\times (1-ir)}$ = $\frac{1-d-ir+d\times ir}{1-d\times ir\times rr-ir+d\times ir}$ = $\frac{1-d-ir+d\times ir}{1-d-ir+d\times ir}$ = 1	
Observed ratios of developing symptoms	$\frac{d\times ir\times rr+(1-d)\times ir}{d\times \left(1-ir\times rr\right)+(1-d)\times (1-ir)}$ = $\frac{d\times ir\times rr+ir-d\times ir}{1-d\times ir\times rr-ir+d\times ir}$ = $\frac{d+ir-d\times ir}{1-d-ir+d\times ir}$	

Equations are derived based on Baratloo et al.’s definitions^11^.

*d* proportions in a population with the disease, *ir* symptom incidence rate, *rr* risk ratios.

In Table 2, a two-symptom case is hypothesized. Two symptoms are associated with the occurrence of the disease. There are incidence rates and risk ratios for presenting both symptoms, one, or none. The correlations between the symptoms can influence the co-occurrence of multiple symptoms and thus joint incidence (*ir*_*both*_) and joint risk ratios (*rr*_*both*_)^2^. The sensitivity and specificity^11^ of presenting two symptoms for the detection of the disease are *ir*_*both*_ × *rr*_*both*_ and *1 − ir*_*both*_ respectively. The sensitivity and specificity^11^ of presenting one of the symptoms for the detection of the disease are *ir*_*one*_ × *rr*_*one*_ and *1 − ir*_*one*_, respectively. When increasing numbers of symptoms present due to the disease status, it becomes more difficult to predict the relationship between the disease status and the symptoms using equations.

**Table 2:** Diagnostic accuracy of two symptoms to diagnose the disease cause in equations.

Disease status	Disease present	Disease absent
Probability	*d*	*1 − d*
Both symptoms present	*d* × *ir*_*both*_ × *rr*_*both*_	(*1 − d*) × *ir*_*both*_
At least one symptoms present	*d* × *ir*_*one*_ × *rr*_*one*_	(*1 − d*) × *ir*_*one*_
Both symptoms absent	*d* × (*1* − *ir*_*both*_ × *rr*_*both*_)	(*1 − d* )× (*1* − *ir*_*both*_ )
At least one symptom absent	*d* × (*1* − *ir*_*one*_ × *rr*_*one*_)	(*1 − d* )× (*1* − *ir*_*one*_ )
**Derived statistics if both symptoms present**		
Sensitivity^11^	(*d* × *ir*_*both*_ × *rr*_*both*_)/*d* = *ir*_*both*_ × *rr*_*both*_	
Specificity^11^	[(*1 − d*) × (*1* − *ir*_*both*_)]/(*1 − d*) = *1* − *ir*_*both*_	
Positive predictive value^11^	(*d* × *ir*_*both*_ × *rr*_*both*_)/[*d* × *ir*_*both*_ × *rr*_*both*_ + (*1 − d*) × *ir*_*both*_] = (*d* × *ir*_*both*_ × *rr*_*both*_)/(*d* × *ir*_*both*_ × *rr*_*both*_ + *ir*_*both*_ − *d* × *ir*_*both*_)	
Negative predictive value^11^	(*1 − d*) × (*1* − *ir*_*both*_)/[*d* × (*1* − *ir*_*both*_ × *rr*_*both*_) + (*1 − d*) × (*1* − *ir*_*both*_)] = (*1 − d* − *ir*_*both*_ + *d* × *ir*_*both*_)/(*1 − d* × *ir*_*both*_ × *rr*_*both*_ − *ir*_*both*_ + *d* × *ir*_*both*_)	
Derived risk ratios of developing 2 symptoms	[*d* × *ir*_*both*_ × *rr*_*both*_ + (*1 − d*) × *ir*_*both*_]/[*d* × (*1* − *ir*_*both*_ × *rr*_*both*_) + (*1 − d*) × (*1* − *ir*_*both*_)] = (*d* × *ir*_*both*_ × *rr*_*both*_ + *ir*_*both*_ − *d* × *ir*_*both*_)/ (*1 − d* × *ir*_*both*_ × *rr*_*both*_ − *ir*_*both*_ + *d* × *ir*_*both*_)	
**Derived statistics if at least one symptom present**		
Sensitivity^11^	(*d* × *ir*_*one*_ × *rr*_*one*_ )/*d* = *ir*_*one*_ × *rr*_*one*_	
Specificity^11^	[(*1 − d*) × (*1* − *ir*_*one*_)]/(*1 − d*) = *1* − *ir*_*one*_	
Positive predictive value^11^	(*d* × *ir*_*one*_ × *rr*_*one*_)/[*d* × *ir*_*one*_ × *rr*_*one*_ + (*1 − d*) × *ir*_*one*_] = (*d* × *ir*_*one*_ × *rr*_*one*_ )/ (*d* × *ir*_*one*_ × *rr*_*one*_ + *ir*_*one*_ − *d* × *ir*_*one*_)	
Negative predictive value^11^	(*1 − d*) × (*1* − *ir*_*one*_)/[*d* × * (1* − *ir*_*one*_ × *rr*_*one*_) + (*1 − d*) × (*1* − *ir*_*one*_)] = (*1 − d* − *ir*_*one*_ + *d* × *ir*_*one*_)/(*1 − d* × *ir*_*one*_ × *rr*_*one*_ − *ir*_*one*_ + *d* × *ir*_*one*_)	

Equations are derived based on Baratloo et al.’s definitions^11^.

*both* both symptoms presenting due to the disease that caused the symptoms, *d* proportions in a population with the disease, *ir* symptom incidence rate, *one* one symptom presenting, *rr* risk ratios.

This study aims to understand the relationships between disease statuses and associated symptoms in terms of their diagnostic test accuracy by simulating populations of various assumed disease prevalence, symptom incidence, risk ratios, and correlations between symptoms.

## Methods

**Figure 1:**
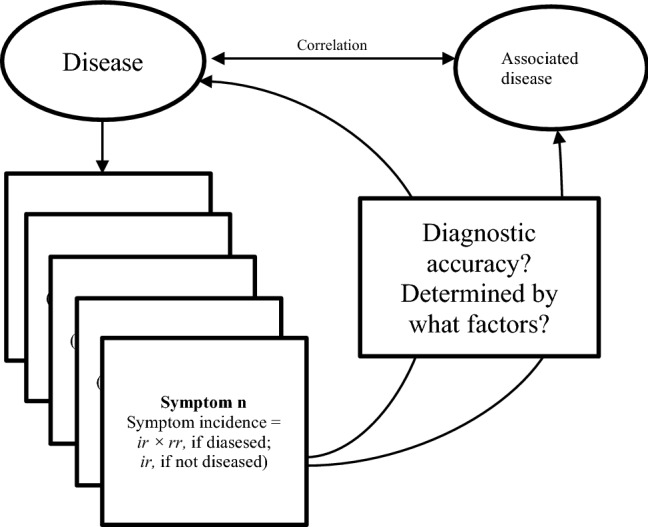
Elements of the simulations in this study. $d$ proportions diseased, $ir$ incidence rate, $rr$ risk ratio.

The assumptions and the epidemiological measures for the simulations are listed in Table 3 and illustrated in Fig. 1. In detail, there were only two diseases. One directly influenced the incidence of the symptoms, and the other was only associated with the disease. The incidence rates and risk ratios were similar with respect to all the simulated symptoms. The products of the incidence rates and risk ratios (at-risk incidence) could not exceed 1, an upper limit of incidence rate of 100%. There were 10,000 individuals simulated each time. There were 40 symptoms induced by the disease. The correlations between the disease and the other associated disease were 0, 0.3, and 0.7. We assumed the prevalence rates of the disease were 0.05, 0.1, 0.2, 0.4, and 0.8, similar to the values adopted in a previous study^2^. Several conditions have been observed in more than 80% of selected populations, for example, Epstein–Barr virus infection^12,13^ and the herpes simplex virus type 2 infection^14^. The baseline incidence of developing symptoms for those not affected by the disease were 0.05, 0.1, 0.2, 0.4, and 0.8. The risk ratios of developing symptoms when diseased were 0.5 (less likely to develop symptoms), 1.0 (equally likely to develop symptoms), 2, 5, 10, and 25 (more likely to develop symptoms). Risk ratios more than 25 were reported in several studies^15–17^. We assumed the correlations between the symptoms were 0, 0.4, and 0.8, similar to a range in a previous study^2^. We assumed the correlations between the diseases were 0, 0.3, and 0.7. We used 10 simulations for each combination of disease correlations, disease prevalence, symptom incidence, symptom correlations, and risk ratios of developing symptoms.

**Table 3:** Assumptions and the assessments of the simulated symptoms.

**Assumptions**		
1	2 diseases of interest: one disease directly related to the symptoms and the other associated with the disease only (unrelated to symptoms)	
2	Similar baseline incidence rates among those not diseased and similar risk ratios for the symptoms	
3	Accurate disease statuses; symptoms reported accurately by patients	
4	The products of baseline incidence rates and risk ratios less than or equal to 1	
5	Similar baseline correlations between symptoms among those diseased or not diseased	
**Epidemiological measures of symptom occurrence in simulations**		
1	Population sizes	10,000
2	Number of symptoms that can be caused by the disease	40
3	Correlations between the disease that caused symptoms and the other associated disease that did not cause symptoms	0, 0.3, and 0.7
4	Prevalence rates or proportions of the population with the disease that caused symptoms	0.05, 0.1, 0.2, 0.4, and 0.8
5	Baseline incidence rates of the symptoms, similar to all symptoms	0.05, 0.1, 0.2, 0.4, and 0.8
6	Risk ratios of developing symptoms if diseased	0.5, 1.0, 2.0, 10.0, and 25.0
7	Correlations between symptoms	0, 0.4, and 0.8
8	Number of simulations for each combination of the above measures 3 to 7	10
**Statistics for assessment**		
	Correlations between symptoms	
	Diagnostic test accuracy (sensitivities, specificities, and AUCs) of the symptoms for the detection of the disease	
	Diagnostic test accuracy (sensitivities, specificities, and AUCs) of the symptoms for the detection of the unrelated disease	

*AUC* area under curve.

### Simulation procedures

The R codes to simulate individuals with assumed epidemiological measures are in Appendix 1. For each simulation, we chose a combination of the above-mentioned epidemiological measures, including disease incidence, associations between diseases, and symptom risk ratios. In a simulation, we created 10,000 individuals and randomly assigned them disease statuses based on the assumed proportions diseased. We also randomly assigned the other associated disease based on its correlations with the main disease using an established method^18,19^. The probability of individuals developing symptoms differed by whether they were diseased or not. Among those diseased, the probability of developing a symptom was the product of its baseline incidence and an assumed risk ratio. Among those not diseased, the probability of developing a symptom was based on the baseline incidence of the symptom, and we created 40 symptoms at the same time. We considered the correlations between the 40 symptoms, and we randomly assigned the symptoms based on disease statuses^18,19^.

### Diagnostic test accuracy of symptoms

We first described the diagnostic accuracy of the symptoms to detect the disease and the other associated disease using equations. Then we used the data obtained from simulations to validate the equations. We defined *sensitivity* as the number of true cases identified by a symptom or symptoms (more than the numbers required by a threshold) divided by the number of those diseased^11,20^. We defined *specificity* as the number of non-cases identified by the absence of a symptom or symptoms (using the same threshold as the sensitivity) divided by the number of those not diseased. The areas under the receiver operating characteristic (ROC) curves and the 95% CIs were derived when using more than one symptom to detect the disease status^21^. We compared the area under curves (AUCs) that were derived from using different numbers of symptoms for disease diagnosis^21^. We chose the best set of sensitivity and specificity in a ROC curve by searching the set with the maximal difference between 1 and the sum of sensitivity and specificity in absolute values^22^. We reported the number of symptoms and the sensitivity and specificity of the best set.

### Approximation of diagnostic accuracy and symptom correlations

We approximated the correlations between symptoms and diagnostic accuracy, including the sensitivities and specificities of single symptoms for disease diagnosis in simulated populations, with epidemiological measures using linear regression. Using linear regression models to approximate complicated measures has been proven to be an effective method to understand the role or importance of various factors on these measures. We considered correlations between symptoms or diagnostic accuracy to be a dependent variable ($Y$), and approximated them by using the above-mentioned epidemiological measures with or without their interaction terms (denoted as ${x}_{i}$, $i$ ranging from 1 to the total number of independent variables in a regression model). The equation was $Y={\alpha }_{0}+{\alpha }_{1}\times {x}_{1}+{\alpha }_{2}\times {x}_{2}+\dots +{\alpha }_{n}\times {x}_{n}$, where ${\alpha }_{0}$ denoted the intercept, ${\alpha }_{i}$ denoted the regression coefficients, and $n$ was the number of independent variables. The implementation of the regression models is available in the R codes in Appendix 1.

We used this approach to interpret principal components^23–25^, determine life stages^26,27^, interpret the diagnosis of frailty syndrome^1^, and demonstrate the biases generated by the diagnostic criteria of mental illnesses^2^. We conduced all the statistical analyses using the R environment (v3.5.1, R Foundation for Statistical Computing, Vienna, Austria)^28^ and RStudio (v1.1.463, RStudio, Inc., Boston, MA)^29^.

## Results

### Quality of simulations and symptom incidence

**Figure 2:**
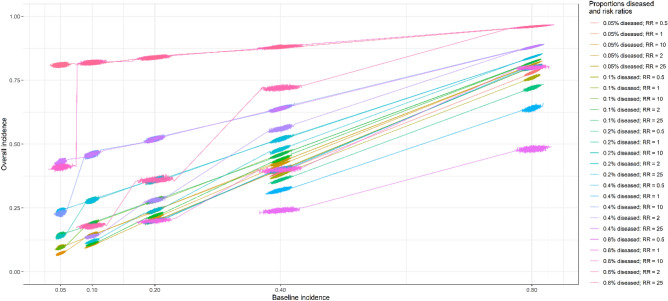
Symptom incidence depending on the baseline incidence, proportions diseased, and risk ratios. *RR* risk ratios.

The derived baseline incidence rates of single symptoms matched the assumed incidence rates, regardless of the assumed proportions diseased, assumed risk ratios, and assumed correlations between symptoms (Appendix 2). The derived risk ratios matched the assumed risk ratios when the at-risk incidence (incidence among those diseased) was less than 1 (Appendix 2). Figure 2 presents the symptom incidence among all subjects. The overall symptom incidence depended on the proportions diseased, baseline symptom incidence, and symptom risk ratios. Based on the similarities between the assumed and derived values, the simulations were well implemented.

### Correlations between symptoms

The correlations between the symptoms ranged from − 0.02 to 0.99 in all simulations (see Table 4). The effects of the assumed epidemiological measures on the correlations between symptoms in the linear regression models depended on whether the at-risk incidence reached 1. The correlations between the two diseases, one causing symptoms and the other only associated with the disease, were not significantly associated with the correlations between the symptoms. The at-risk incidence reaching 1 or not, proportions diseased, risk ratios, at-risk incidence, and symptom correlations among those not diseased (baseline symptom correlations) were significantly and positively associated with the overall symptom correlations. The baseline incidence was negatively and significantly associated with the overall symptom correlations. The adjusted R-squared was 0.86 and 0.89 with at-risk incidence reaching 1 or not, respectively.

**Table 4:** Effects of baseline incidence, proportions diseased, risk ratios, and baseline symptom correlations on overall symptom correlations.

	Coefficients	(95% CIs)	*p*
**Incidence not reaching 1 among those diseased**			
(Intercept)	− 0.007	(− 0.008 to − 0.006)	< 0.0001
Correlation between diseases	0	(− 0.001 to 0.001)	0.859
Proportions diseased	0.119	(0.117 to 0.121)	< 0.0001
Baseline incidence	− 0.107	(− 0.109 to − 0.105)	< 0.0001
Risk ratio	0.016	(0.016 to 0.016)	< 0.0001
At-risk incidence	0.185	(0.183 to 0.187)	< 0.0001
Baseline symptom correlation	0.841	(0.840 to 0.842)	< 0.0001
Dependent variables mean = 0.5; SD = 0.31; median = 0.47; min = − 0.04; max = 0.99			
Adjusted R-square = 0.89			
**Incidence reaching 1 among those diseased**			
(Intercept)	0.339	(0.336 to 0.342)	< 0.0001
Correlation between diseases	0	(− 0.002 to 0.002)	0.736
Proportions diseased	0.315	(0.312 to 0.318)	< 0.0001
Baseline incidence	− 0.337	(− 0.341 to − 0.333)	< 0.0001
Risk ratio	0.01	(0.01 to 0.01)	< 0.0001
Baseline symptom correlation	0.639	(0.637 to 0.641)	< 0.0001
Dependent variables mean = 0.62; SD = 0.29; median = 0.72; min = − 0.02; max = 0.99			
Adjusted R-square = 0.86			

*CI* confidence interval, *SD* standard deviation.

### Diagnostic test accuracy of individual symptoms for the detection of the diseases

As expected in Table 1, the sensitivities and specificities of individual symptoms for disease diagnosis can be predicted with at-risk incidence (Table 5) and 1 minus baseline incidence (Table 6), respectively. The sensitivities of individual symptoms for disease diagnosis were 1 for all symptoms, when the at-risk incidence reached 1. The effect sizes of disease correlations, proportions diseased, risk ratios, and baseline symptom correlations remained the same, when the at-risk incidence reached 1 or not.

**Table 5:** Effects of baseline incidence, proportions diseased, risk ratios, and baseline symptom correlations on the sensitivities of individual symptoms for disease diagnosis.

	Coefficients	(95% CIs)	*p*
**Incidence not reaching 1 among those diseased**			
(Intercept)	0	(0 to 0)	< 0.0001
Correlation between diseases	0	(0 to 0)	0.82
Proportions diseased	0	(0 to 0)	0.03
Baseline incidence	0	(0 to 0)	< 0.0001
Risk ratio	0	(0 to 0)	< 0.0001
At-risk incidence	1	(1 to 1)	< 0.0001
Baseline symptom correlation	0	(0 to 0)	0.22
Dependent variable mean = 0.64; SD = 0.32; median = 0.55; min = 0.12; max = 1			
Adjusted R-square = 1			
**Incidence reaching 1 among those diseased**			
(Intercept)	1	(1 to 1)	< 0.0001
Correlation between diseases	0	(0 to 0)	0.24
Proportions diseased	0	(0 to 0)	0.34
Baseline incidence	0	(0 to 0)	0.22
Risk ratio	0	(0 to 0)	0.71
Baseline symptom correlation	0	(0 to 0)	0.22
Dependent variable mean = 1; SD = 0; median = 1; min = 1; max = 1			
Adjusted R-square = 0.5			

*CI* confidence interval, *SD* standard deviation.

**Table 6:** Effects of baseline incidence, proportions diseased, risk ratios, and baseline symptom correlations on the specificities of individual symptoms for disease diagnosis.

	Coefficients	(95% CIs)	*p*
**Incidence not reaching 1 among those diseased**			
(Intercept)	1	(1 to 1)	< 0.0001
Correlation between diseases	0	(0 to 0)	0.03
Proportions diseased	0	(0 to 0)	0.01
Baseline incidence	− 1	(− 1 to − 1)	< 0.0001
Risk ratio	0	(0 to 0)	< 0.0001
At-risk incidence	0	(0 to 0)	< 0.0001
Baseline symptom correlation	0	(0 to 0)	0.1
Dependent variable mean = 0.62; SD = 0.27; median = 0.61; min = 0.17; max = 0.97			
Adjusted R-square = 1			
**Incidence reaching 1 among those diseased**			
(Intercept)	1	(1 to 1)	< 0.0001
Correlation between diseases	0	(0 to 0)	0.95
Proportions diseased	0	(0 to 0)	0.24
Baseline incidence	− 1	(− 1 to − 1)	< 0.0001
Risk ratio	0	(0 to 0)	< 0.0001
Baseline symptom correlation	0	(0 to 0)	0.97
Dependent variable mean = 0.61; SD = 0.29; median = 0.63; min = 0.17; max = 0.97			
Adjusted R-square = 1			

*CI* confidence interval, *SD* standard deviation.

### Diagnostic test accuracy of symptom numbers for disease diagnosis

**Figure 3:**
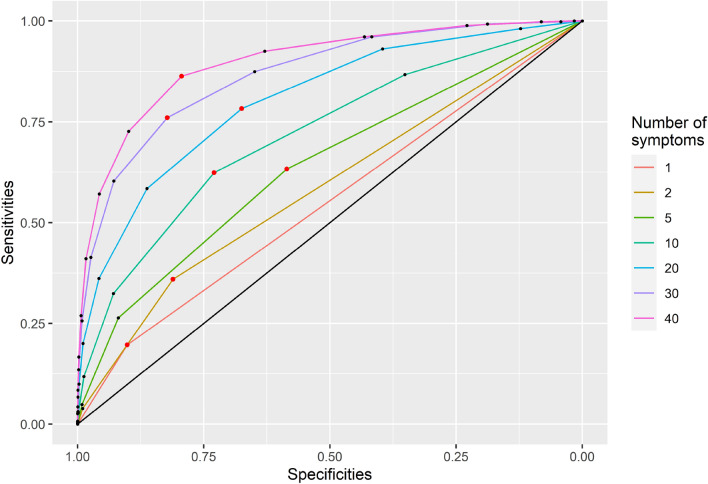
Receiver operating characteristic (ROC) curves for disease diagnosis based on the numbers of symptoms. Red dots = the set of sensitivities and specificities with the largest difference in the absolute values between 1 and the sums of sensitivities and specificities in a ROC curve. For each number of symptoms used for disease diagnosis, one red dot—a best set of sensitivities and specificities—was selected. With a maximum of 40 symptoms used for disease diagnosis, ROC curves in this figure were created assuming the risk ratio as 2, baseline symptom incidence as 0.1, proportions diseased as 0.05, no correlations between diseases, and no correlations between symptoms.

When using the accumulative numbers of symptoms to predict the disease directly causing the symptoms, we used the AUCs to compare the diagnostic test accuracy across the numbers of symptoms used. Figure 3 shows one example of the ROC curve assuming the risk ratio as 2, baseline symptom incidence as 0.1, proportions diseased as 0.05, no correlations between diseases, and no correlations between symptoms. When more symptoms were used for disease diagnosis, the AUCs increased. We selected the best set of sensitivities and specificities for disease diagnosis based on the sums of sensitivities and specificities (red dots in Fig. 3). The red dots also represent the diagnostic thresholds for disease diagnosis. For example, when using 40 symptoms for disease diagnosis, the threshold of obtaining the best set of sensitivities and specificities was 5.5 (see the red dots in Fig. 3). This result suggested that when there were 6 or more symptoms out of 40 presenting in individual patients, the sensitivity to detect the disease cause was 86.3%. The specificity for correctly excluding the disease in individuals with less than 6 symptoms out of 40 was 79.4%. For other combinations of epidemiologic measures, see examples in Appendix 3.

Table 7 shows the effects of epidemiological measures on the AUCs of individual symptoms for disease diagnosis. The AUCs of individual symptoms can be explained fully by disease correlations, proportions diseased, baseline symptom incidence, risk ratios, at-risk symptom incidence, and symptom correlations (adjusted R-squared = 1 for at-risk incidence reaching 1 or not). When the at-risk incidence was less than 1, the at-risk incidence and baseline incidence had the same effect sizes of opposite directions, a regression coefficient of 0.5 and − 0.5, respectively. When the at-risk incidence reached 1, the AUCs of individual symptoms decreased with the baseline symptom incidence (regression coefficient = − 0.5) from 1 (perfect diagnostic accuracy).

**Table 7:** Effects of baseline incidence, proportions diseased, risk ratios, and baseline symptom correlations on the area under the receiver operating characteristic curve of individual symptoms for disease diagnosis.

	Coefficients	(95% CIs)	*p*
**Incidence not reaching 1 among those diseased**			
(Intercept)	0.5	(0.5 to 0.5)	< 0.0001
Correlation between diseases	0	(0 to 0)	0.25
Proportions diseased	0	(0 to 0)	0.57
Baseline incidence	− 0.5	(− 0.5 to − 0.5)	< 0.0001
Risk ratio	0	(0 to 0)	< 0.0001
At-risk incidence	0.5	(0.5 to 0.5)	< 0.0001
Baseline symptom correlation	0	(0 to 0)	0.44
Dependent variable mean = 0.63; SD = 0.27; median = 0.6; min = 0.27; max = 0.98			
Adjusted R-square = 1			
**Incidence reaching 1 among those diseased**			
(Intercept)	1	(1 to 1)	< 0.0001
Correlation between diseases	0	(0 to 0)	0.21
Proportions diseased	0	(0 to 0)	0.37
Baseline incidence	− 0.5	(− 0.5 to − 0.5)	< 0.0001
Risk ratio	0	(0 to 0)	< 0.0001
Baseline symptom correlation	0	(0 to 0)	0.06
Dependent variable mean = 0.8; SD = 0.15; median = 0.8; min = 0.59; max = 0.98			
Adjusted R-square = 1			

*CI* confidence interval, *SD* standard deviation.

In Table 8, using a maximum of 40 symptoms for disease diagnosis, we analyzed the effects of the epidemiological measures on the observed maximal AUCs. The effect sizes and statistical significance of the epidemiologic measures depended on whether the at-risk incidence reached 1. The correlations between diseases were not significant (*p* > 0.68 for both). Proportions diseased were significantly and positively associated with the maximal AUCs (*p* < 0.05 for all). Baseline symptom incidence and symptom correlations were significantly and negatively associated with the maximal AUCs (*p* < 0.05 for all). The maximal AUCs can be well predicted by epidemiologic measures when the at-risk incidence reached 1 or not (adjusted R-squared = 0.83 and 0.80, respectively).

**Table 8:** Effects of baseline incidence, proportions diseased, risk ratios, and baseline symptom correlations on the maximal area under the receiver operating characteristic curve using at most 40 symptoms for disease diagnosis.

	Coefficients	(95% CIs)	*p*
**Incidence not reaching 1 among those diseased**			
(Intercept)	0.58	(0.569 to 0.591)	< 0.0001
Correlation between diseases	− 0.001	(− 0.012 to 0.01)	0.807
Proportions diseased	0.006	(− 0.006 to 0.018)	0.322
Baseline incidence	− 0.763	(− 0.781 to − 0.745)	< 0.0001
Risk ratio	− 0.013	(− 0.014 to − 0.012)	< 0.0001
At-risk incidence	0.775	(0.762 to 0.788)	< 0.0001
Baseline symptom correlation	− 0.104	(− 0.114 to − 0.094)	< 0.0001
Dependent variable mean = 0.7; SD = 0.29; median = 0.76; min = 0; max = 1			
Adjusted R-square = 0.79			
**Incidence reaching 1 among those diseased**			
(Intercept)	1.125	(1.116 to 1.134)	< 0.0001
Correlation between diseases	0.004	(− 0.004 to 0.012)	0.303
Proportions diseased	− 0.007	(− 0.015 to 0.001)	0.088
Baseline incidence	− 0.256	(− 0.268 to − 0.244)	< 0.0001
Risk ratio	− 0.003	(− 0.004 to − 0.002)	< 0.0001
Baseline symptom correlation	− 0.177	(− 0.184 to − 0.17)	< 0.0001
Dependent variable mean = 0.94; SD = 0.1; median = 0.99; min = 0.66; max = 1			
Adjusted R-square = 0.71			

*CI* confidence interval, *SD* standard deviation.

**Figure 4:**
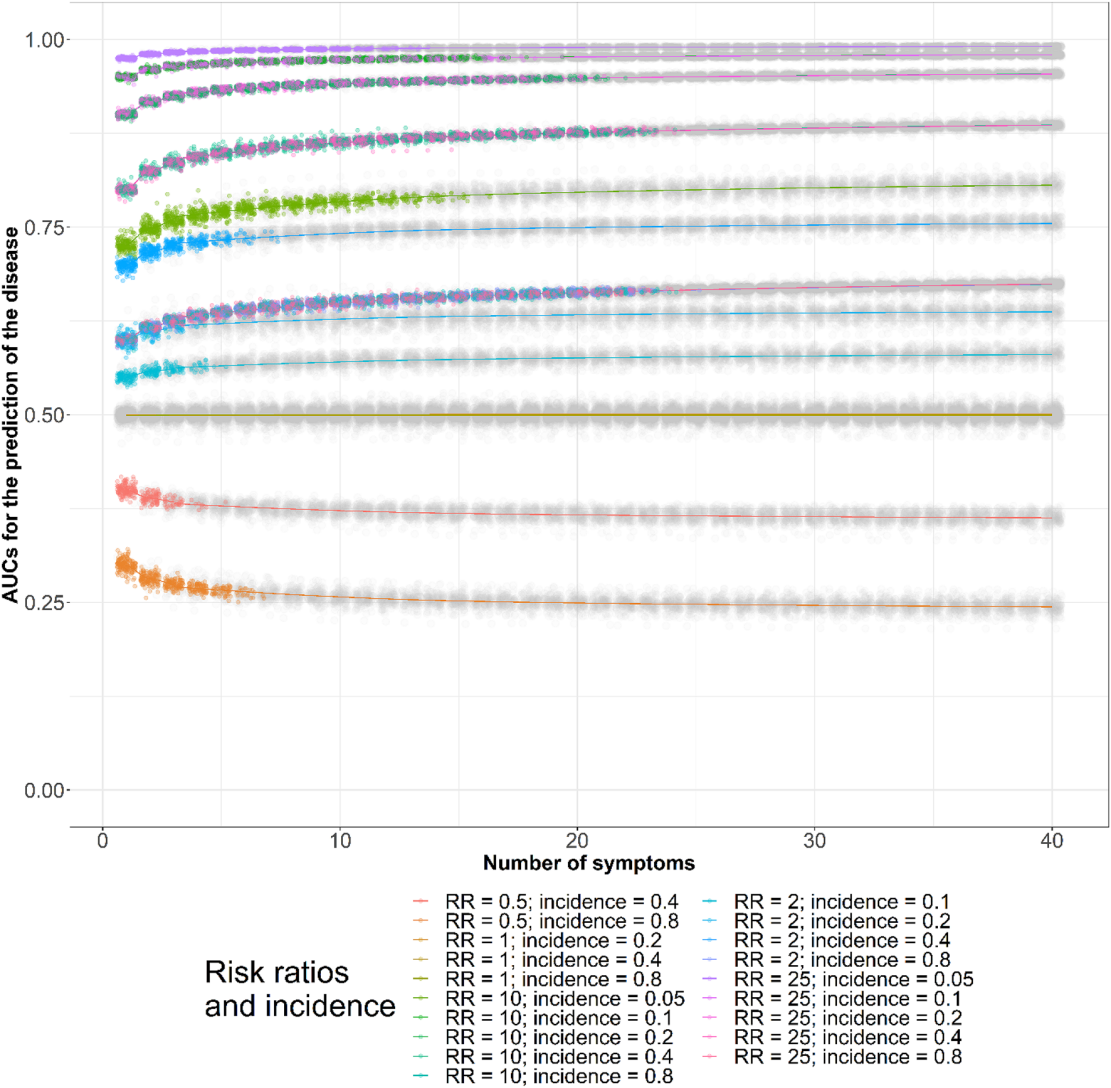
Areas under the receiver operating characteristic curves for disease diagnosis by numbers of symptoms, baseline symptom incidence, and symptom risk ratios. *AUC* area under curve, *CI* confidence interval, *RR* risk ratio, *incidence* baseline symptom incidence among those not diseased. Gray dots are the area under curve (AUCs) whose 95% confidence intervals (CIs) overlapped with the maximal AUC 95% CIs identified using a maximum of 40 symptoms for disease diagnosis. The lines were added to show the AUCs assuming the same epidemiological measures. All AUCs assuming 0.8 correlations between symptoms among those not diseased are illustrated.

Figure 4 presents the changes in the AUCs according to the numbers of symptoms used for disease diagnosis using simulations assuming 0.8 correlations between symptoms among those not diseased. We colored the AUCs based on the observed risk ratios and baseline symptom incidence. In each simulation, when the 95% CIs of the AUCs overlapped those of the maximal or minimal AUCs with risk ratios greater or less than 1, respectively, we colored the dots gray. The AUCs changed when we used more symptoms for disease diagnosis. In Fig. 4, the 95% CIs of all of the AUCs in the simulations assuming risk ratios as 1 overlapped with the 95% CIs of the maximal or minimal AUCs. The AUCs in the simulations assuming 0 and 0.4 symptom correlations among individuals not diseased are presented in Appendix 4.

**Figure 5:**
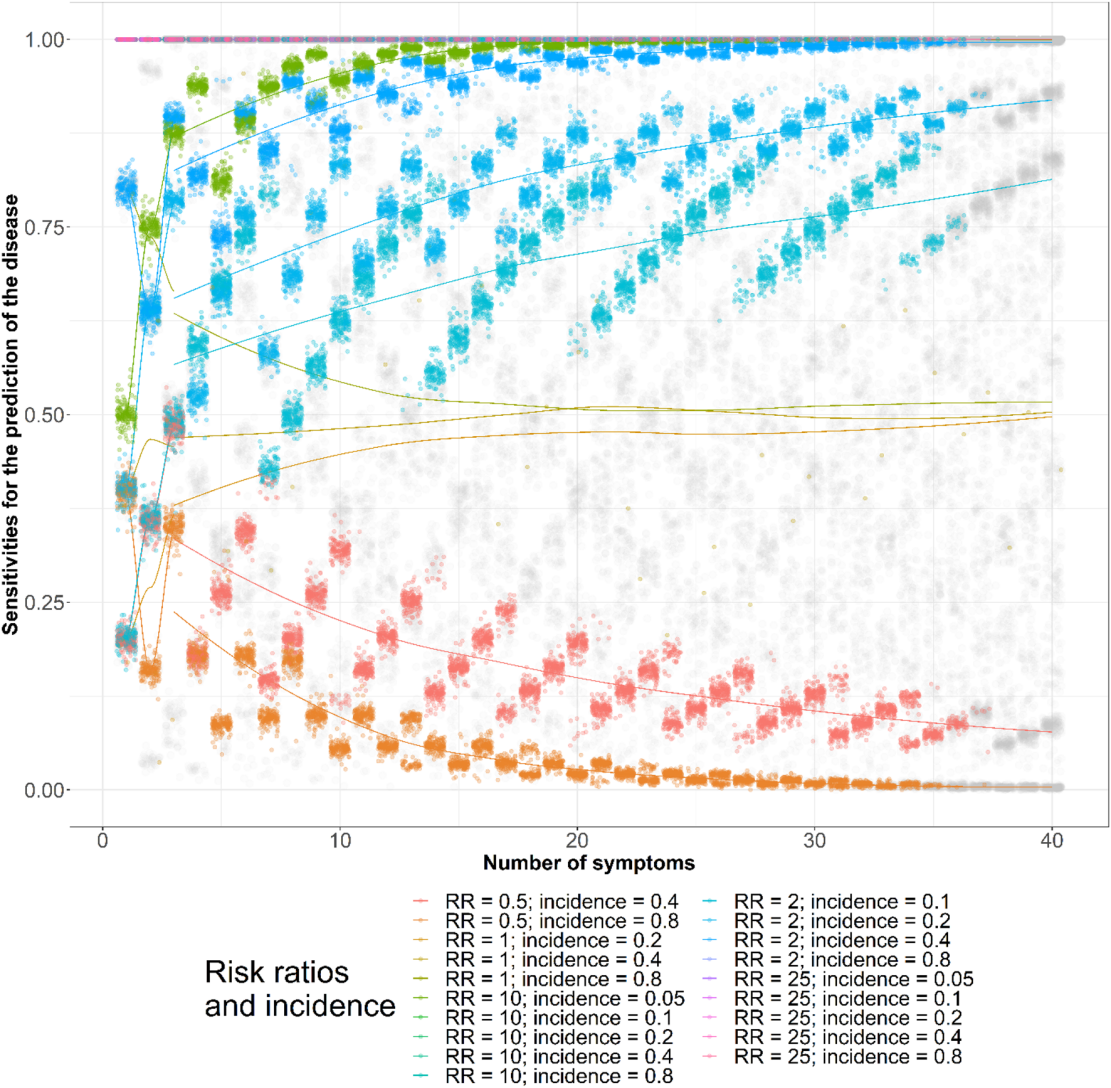
Sensitivities for disease diagnosis by numbers of symptoms, baseline symptom incidence, and symptom risk ratios. *AUC* area under curve, *CI* confidence interval, *RR* risk ratio, *incidence* baseline symptom incidence among those not diseased. Gray dots are the area under curve (AUCs) whose 95% confidence intervals (CIs) overlapped with the maximal AUC 95% CIs identified using a maximum of 40 symptoms for disease diagnosis. The lines were added to show the AUCs assuming the same epidemiological measures. All AUCs assuming 0.8 correlations between symptoms among those not diseased are illustrated.

**Figure 6:**
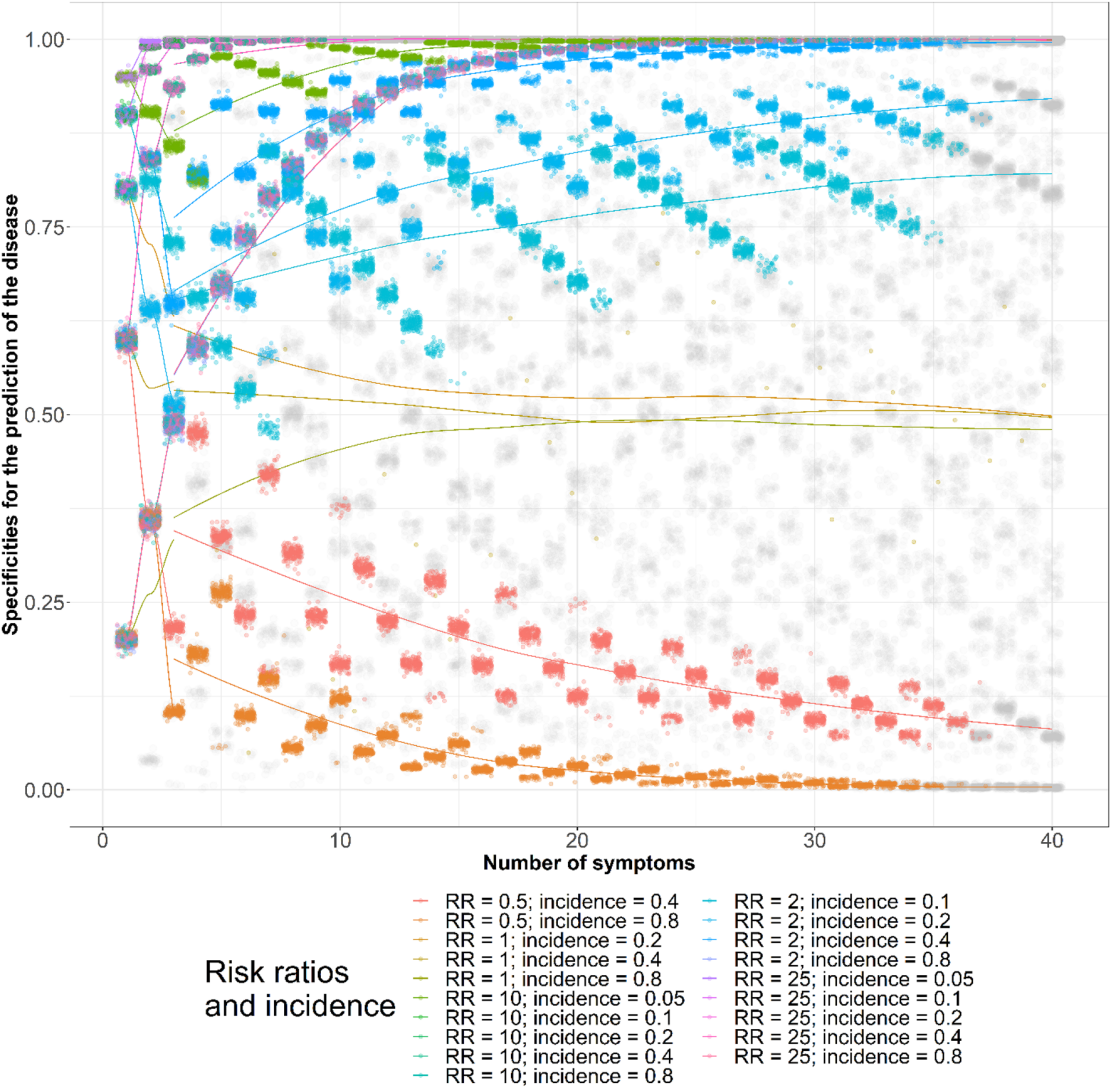
Specificities for disease diagnosis by numbers of symptoms, baseline symptom incidence, and symptom risk ratios. *AUC* area under curve, *CI* confidence interval, *RR* risk ratio, *incidence* baseline symptom incidence among those not diseased. Gray dots are the area under curve (AUCs) whose 95% confidence intervals (CIs) overlapped with the maximal AUC 95% CIs identified using a maximum of 40 symptoms for disease diagnosis. The lines were added to show the AUCs assuming the same epidemiological measures. All AUCs assuming 0.8 correlations between symptoms among those not diseased are illustrated.

The best sets of sensitivities and specificities for disease diagnosis chosen based on the AUCs are plotted in Figs. 5 and 6, respectively. We plotted the sensitivities and specificities according to the assumed risk ratios and baseline symptom incidence. When the 95% CIs of the AUCs overlapped with the 95% CIs of the maximal AUCs, we colored the dots gray. The role of the epidemiologic measures in the best sets of sensitivities and specificities are listed in Tables 9 and 10, respectively.

**Table 9:** Effects of baseline incidence, proportions diseased, risk ratios, and baseline symptom correlations on the sensitivities obtained from the maximal area under the receiver operating characteristic curve using at most 40 symptoms for disease diagnosis.

	Coefficients	(95% CIs)	*p*
**Incidence not reaching 1 among those diseased**			
(Intercept)	0.394	(0.38 to 0.408)	< 0.0001
Correlation between diseases	− 0.003	(− 0.017 to 0.011)	0.664
Proportions diseased	0.001	(− 0.014 to 0.016)	0.853
Baseline incidence	− 0.49	(− 0.512 to − 0.468)	< 0.0001
Risk ratio	− 0.008	(− 0.009 to − 0.007)	< 0.0001
At-risk incidence	0.879	(0.863 to 0.895)	< 0.0001
Baseline symptom correlation	− 0.082	(− 0.095 to − 0.069)	< 0.0001
Dependent variable mean = 0.71; SD = 0.32; median = 0.81; min = 0; max = 1			
Adjusted R-square = 0.72			
**Incidence reaching 1 among those diseased**			
(Intercept)	1	(1 to 1)	< 0.0001
Correlation between diseases	0	(0 to 0)	0.26
Proportions diseased	0	(0 to 0)	0.353
Baseline incidence	0	(0 to 0)	0.244
Risk ratio	0	(0 to 0)	0.725
Baseline symptom correlation	0	(0 to 0)	0.222
Dependent variable mean = 1; SD = 0; median = 1; min = 1; max = 1			
Adjusted R-square = 0.5			

*CI* confidence interval, *SD* standard deviation.

**Table 10:** Effects of baseline incidence, proportions diseased, risk ratios, and baseline symptom correlations on the specificities obtained from the maximal area under the receiver operating characteristic curve using at most 40 symptoms for disease diagnosis.

	Coefficients	(95% CIs)	*p*
**Incidence not reaching 1 among those diseased**			
(Intercept)	0.72	(0.706 to 0.734)	< 0.0001
Correlation between diseases	0	(− 0.014 to 0.014)	0.968
Proportions diseased	0.01	(− 0.005 to 0.025)	0.201
Baseline incidence	− 0.899	(− 0.921 to − 0.877)	< 0.0001
Risk ratio	− 0.013	(− 0.014 to − 0.012)	< 0.0001
At-risk incidence	0.616	(0.6 to 0.632)	< 0.0001
Baseline symptom correlation	− 0.116	(− 0.129 to − 0.103)	< 0.0001
Dependent variable mean = 0.69; SD = 0.29; median = 0.77; min = 0; max = 1			
Adjusted R-square = 0.69			
**Incidence reaching 1 among those diseased**			
(Intercept)	1.249	(1.23 to 1.268)	< 0.0001
Correlation between diseases	0.008	(− 0.008 to 0.024)	0.33
Proportions diseased	− 0.015	(− 0.032 to 0.002)	0.084
Baseline incidence	− 0.511	(− 0.535 to − 0.487)	< 0.0001
Risk ratio	− 0.005	(− 0.006 to − 0.004)	< 0.0001
Baseline symptom correlation	− 0.354	(− 0.368 to − 0.34)	< 0.0001
Dependent variable mean = 0.88; SD = 0.2; median = 0.98; min = 0.33; max = 1			
Adjusted R-square = 0.71			

*CI* confidence interval, *SD* standard deviation.

In Table 9, the best sets of sensitivities chosen based on the maximal or minimal AUCs—when the risk ratios were greater or less than 1, respectively—were approximated with epidemiological measures. When the at-risk incidence reached 1, the sensitivities were 1, and the epidemiological measures were not significantly associated with the sensitivities. The correlations between diseases and proportions diseased were not significant when the at-risk incidence did not reach 1 (*p* > 0.05 for both). The baseline symptom incidence, risk ratios, and baseline symptom correlations were negatively associated with the best-set sensitivities when the at-risk incidence was less than 1 (*p* < 0.0001 for all). The variances of the best-set sensitivities can be explained mostly by epidemiological measures when the at-risk incidence was less than 1 (adjusted R-squared = 0.72).

In Table 10, the best sets of specificities chosen based on maximal or minimal AUCs—when the risk ratios were greater or less than 1, respectively—were approximated with epidemiological measures. The correlations between diseases and proportions diseased were not significantly associated with the best-set specificities when the at-risk incidence reached 1 or not (*p* > 0.05 for all). When the at-risk incidence was less than 1, the at-risk incidence was positively and significantly associated with specificities (*p* < 0.05). The baseline symptom incidence, risk ratios, and baseline symptom correlations were negatively and significantly associated with specificities, and the effect sizes depended on whether the at-risk incidence reached 1 (*p* < 0.0001 for all). The adjusted R-squared was 0.71 and 0.69 when the at-risk incidence reached 1 or not, respectively.

### Diagnostic accuracy for the disease associated with the disease causing symptoms

The diagnostic accuracy for the disease associated with the disease causing symptoms were approximated with the epidemiological measures shown in Table 11. When the at-risk incidence was less than 1, the correlations between the diseases and their interaction terms with baseline symptom incidence, risk ratios, and baseline symptom correlations were significantly associated with the AUCs to predict the associated disease (*p* < 0.0001 for all). The main effects of baseline symptom incidence, risk ratios, and at-risk incidence also were significant (*p* < 0.0001 for all). When the at-risk incidence reached 1, the correlations between the diseases and their interaction terms with the baseline symptom incidence, risk ratios, and baseline symptom correlations remained significantly associated with the AUCs to predict the associated disease (*p* < 0.0001 for all). The proportions of the AUC variances explained by the epidemiological measures depended on whether the at-risk incidence reached 1 or not, adjusted R-squared = 0.96 and 0.66, respectively.

**Table 11:** Effects of the correlations between diseases, baseline incidence, proportions diseased, risk ratios, and baseline symptom correlations on the areas under curves obtained from the maximal area under the receiver operating characteristic curve using at most 40 symptoms to predict the disease associated with the disease that caused symptoms.

	Coefficients	(95% CIs)	*p*
**Incidence not reaching 1 among those diseased**			
(Intercept)	0.462	(0.453 to 0.471)	< 0.0001
Correlation between diseases	0.208	(0.187 to 0.229)	< 0.0001
Proportions diseased	− 0.002	(− 0.013 to 0.009)	0.681
Baseline incidence	− 0.195	(− 0.21 to − 0.18)	< 0.0001
Risk ratio	− 0.014	(− 0.015 to − 0.013)	< 0.0001
At-risk incidence	0.262	(0.254 to 0.27)	< 0.0001
Baseline symptom correlation	− 0.005	(− 0.014 to 0.004)	0.265
Correlation between diseases:Proportions diseased	0.009	(− 0.016 to 0.034)	0.496
Correlation between diseases:Baseline incidence	− 0.197	(− 0.228 to − 0.166)	< 0.0001
Correlation between diseases:Risk ratio	0.027	(0.025 to 0.029)	< 0.0001
Correlation between diseases:Baseline symptom correlation	− 0.094	(− 0.116 to − 0.072)	< 0.0001
Dependent variable mean = 0.57; SD = 0.14; median = 0.51; min = 0.13; max = 0.88			
Adjusted R-square = 0.66			
**Incidence reaching 1 among those diseased**			
(Intercept)	0.508	(0.502 to 0.514)	< 0.0001
Correlation between diseases	0.618	(0.604 to 0.632)	< 0.0001
Proportions diseased	− 0.007	(− 0.013 to − 0.001)	0.025
Baseline incidence	0	(− 0.008 to 0.008)	0.973
Risk ratio	0	(0 to 0)	0.764
Baseline symptom correlation	− 0.003	(− 0.008 to 0.002)	0.174
Correlation between diseases:Proportions diseased	0.002	(− 0.011 to 0.015)	0.774
Correlation between diseases:Baseline incidence	− 0.254	(− 0.273 to − 0.235)	< 0.0001
Correlation between diseases:Risk ratio	− 0.002	(− 0.003 to − 0.001)	< 0.0001
Correlation between diseases:Baseline symptom correlation	− 0.182	(− 0.193 to − 0.171)	< 0.0001
Dependent variable mean = 0.65; SD = 0.13; median = 0.64; min = 0.47; max = 0.88			
Adjusted R-square = 0.96			

*CI* confidence interval, *SD* standard deviation.

### Observed symptom correlations and incidence on the AUCs

In Table 12, the AUCs to predict the disease directly causing symptoms were approximated with observable measures: overall symptom correlations, overall symptom incidence, and numbers of symptoms used for disease diagnosis. The overall symptom correlations and numbers of symptoms were positively and significantly associated with AUCs for disease diagnosis (coefficients = 0.145 and 0.001, respectively; *p* < 0.0001 for both). The overall symptom incidence was negatively and significantly associated with AUCs (coefficient =  − 0.033, *p* < 0.0001). However, these three measures only explained a small fraction of the AUC variances for all risk ratios or when the risk ratios were greater than 1, adjusted R-squared = 0.03 and 0.02, respectively.

**Table 12:** Role of numbers of symptoms, overall symptom correlations, and overall symptom incidence on the AUCs for disease diagnosis.

	Coefficients	(95% CIs)	*p*
**All RRs**			
(Intercept)	0.667	(0.664 to 0.67)	< 0.0001
Overall symptom correlation	0.145	(0.142 to 0.148)	< 0.0001
Overall symptom incidence	− 0.033	(− 0.036 to − 0.03)	< 0.0001
Number of symptoms	0.001	(0.001 to 0.001)	< 0.0001
Dependent variable mean = 0.75; SD = 0.26; median = 0.84; min = 0; max = 1			
Adjusted R-square = 0.03			
**RRs > 1**			
(Intercept)	0.803	(0.801 to 0.805)	< 0.0001
Overall symptom correlation	0.02	(0.018 to 0.022)	< 0.0001
Overall symptom incidence	− 0.008	(− 0.01 to − 0.006)	< 0.0001
Number of symptoms	0.002	(0.002 to 0.002)	< 0.0001
Dependent variable mean = 0.75; SD = 0.26; median = 0.84; min = 0; max = 1			
Adjusted R-square = 0.02			

*CI* confidence interval.

## Discussions

This is the first study to estimate the diagnostic accuracy of single symptoms and the numbers of symptoms, based on simulations that have been used to demonstrate the biases in the diagnostic criteria of mental illnesses^2^. When single symptoms are caused by a common disease and used to predict disease status, the sensitivities and specificities of single symptoms can be predicted fully with the at-risk incidence and 1 minus baseline symptom incidence, respectively. This can be proved by mathematical equations or observed in simulations. However, when two or more symptoms of the same disease cause are used to estimate disease status, the estimates of the joint incidence rates, joint risk ratios, and joint at-risk incidence are required in the equations describing these multiple symptoms. Therefore, it becomes complicated to derive diagnostic accuracy in mathematical equations, and so it is practical to estimate the diagnostic accuracy of multiple symptoms through simulations. Key epidemiological measures for symptom development were identified in the equations: proportions diseased, baseline symptom incidence, and risk ratios of symptom development. The correlations between symptoms are important when more than one symptom are used for disease diagnosis. A combination of these epidemiological measures of the symptoms can be used to simulate symptom development according to disease status. When at most two symptoms occur in a population, the diagnostic accuracy—sensitivities and specificities—of having 0, 1, and 2 symptoms can be derived to construct a ROC and its AUC. By repeating this process until 40 symptoms are used, the AUCs increase or decrease or remain around 0.5 when risk ratios are greater than 1, less than 1, or equals 1, respectively. For a combination of the epidemiological measures, the maximal AUCs can be selected from the simulations. We selected the best sets of sensitivities and specificities whose absolute values had the largest differences between their sums and 1, for a given AUC. The trade-off between sensitivities and specificities can be observed^1^, when more symptoms are used for disease diagnosis.

For a combination of epidemiological measures, AUCs tend to reach the plateau with less than 30 symptoms, particularly when baseline symptom correlations are closer to 0, i.e., symptoms are not statistically correlated. The maximal AUCs can be well approximated with baseline incidence, risk ratios, at-risk incidence, and baseline symptom correlations (adjusted R-squared > 0.71). The best sets of sensitivities and specificities also can be well approximated with these measures (adjusted R-squared > 0.69). However, in the real world, symptom incidence and risk ratios cannot be determined when the disease status cannot be precisely confirmed. We found that the three observable measures—overall symptom correlations, overall symptom incidence, and numbers of symptoms—do not well explain the AUC variances (adjusted R-squared = 0.03). When researchers are confident that the RRs are greater than 1 (AUCs increase with the numbers of symptoms), the observable measures explain the AUC variances even worse (adjusted R-squared = 0.02).

### Evidence-based recommendations?

A previous study has provided several recommendations for how to use age-related symptoms to diagnose a geriatric syndrome, frailty^7^. The first recommendation for using symptoms for frailty diagnosis was to explicitly select these symptoms based on their associations with health status^7^. The authors did not provide recommendations about selecting symptoms directly associated with frailty^7^. The second recommendation was to choose symptoms that become more prevalent with age^7^. The third recommendation was to choose symptoms that do not saturate early in the life stage (do not become very prevalent among the elderly)^7^. The fourth recommendation was to include symptoms developed from different systems^7^, for example, not to include only symptoms related to changes in cognition^7^. The last recommendation was to use the same frailty indices consisting of the same symptoms, when the indices are used in the same populations in different time points^7^. The authors thought different frailty indices often yield similar results in the same samples^7.^ One additional recommendation was to use at least 30 to 40 symptoms to create frailty indices, since they claimed that using more symptoms leads to more precise estimates^7^.

No scientific evidence exists to support the first three above-mentioned recommendations^7^. In fact, these three recommendations are likely to contradict our findings. When symptoms were used to predict a disease not directly associated with them in our simulations, the diagnostic accuracy of the symptoms for the associated disease partly depended on the correlations between the associated disease and the disease that directly caused symptoms (Table 11). When health-related symptoms are chosen based on health status and used to predict frailty, the correlations between health status and frailty should be well determined to understand their role in the diagnostic accuracy of the health-related symptoms for frailty. The first recommendation failed to recognize that the diagnostic accuracy of the health-related symptoms for frailty diagnosis depends on the correlations between health status and frailty and their interaction terms with baseline symptom incidence, risk ratios, and baseline symptom correlations.

The second and third recommendations require the symptoms to also be associated with age^7^. In addition to being caused by frailty in theory, the symptoms used to predict frailty are required to be associated with both health status and age. This approach creates a causal network that is difficult to simulate due to the large number of epidemiological measures involved, including the associations between age, health status, and frailty (3 parameters), how they interact with the baseline incidence and risk ratios of symptoms (3 X 2 parameters), and many others. This complexity is beyond what our simulations could handle and thus further evidence to justify these recommendations would be required. However, to our knowledge, no clear evidence exists to support the hypothesized casual network associated with these two recommendations.

The second and third recommendations also impose limits on the prevalence of the symptoms for frailty diagnosis^7^. The prevalence of frailty symptoms could not be too low because they need to increase with age according to the second recommendation^7^. Frailty symptoms could not be too common so that they would not saturate early^7^. In our simulations, overall symptom incidence failed to explain a large proportion of AUC variances, and was, in fact, negatively associated with diagnostic accuracy, AUCs. When baseline symptom incidence (among those not diseased only) can be estimated, it is negatively associated with the specificities of individual symptoms. We do not have sufficient evidence to support the recommendations to select frailty symptoms based on overall symptom prevalence.

Our findings partly address the fourth recommendation that encourages using symptoms from various human systems. Baseline symptom correlations (among those not diseased) are significantly and negatively associated with the maximal AUCs, when the at-risk incidence among those diseased reached 1 or not. This recommendation may make better sense, particularly when symptoms from various human systems are less correlated. In the simulations, overall symptom correlations that are observable are significantly and positively associated with AUCs, though slightly. It is unclear whether the ranges of correlations that the recommendation authors aimed to suggest and this recommendation can be improved based on our findings.

The additional recommendation that encourages using more symptoms (at least 30) for disease diagnosis is not supported by any evidence^7^. Our simulations show that diagnostic accuracy measured with AUCs often reaches a plateau at 30 or fewer symptoms. Moreover, the frailty indices produced by the authors of the recommendations being discussed have been criticized for using an excessive number of symptoms^1^. Their frailty indices seem overcomplicated and can be simplified with fewer symptoms, because many of the input symptoms are correlated^1^.

### Implications for the use of diagnostic criteria

Currently the diagnosis of many conditions, such as mental illnesses^2,30^ and frailty indices^1,9^, are based on composite diagnostic criteria. Both mental illnesses and frailty indices use symptoms to confirm diagnoses^1,2^. However, recently several issues related to composite diagnostic criteria have been identified. The most important issue is that complicated diagnostic criteria introduce biases into the diagnoses^1,31^. The input symptoms often are summed and censored with certain thresholds to derive intermediate variables or confirm diagnoses^1^. When the numbers or sum of symptoms are censored, biases that are not explained by the input symptoms can be generated and introduced to the diagnoses^1^. Therefore, the diagnoses of frailty have poor relationships with the input symptoms and do not predict major outcomes better than their input symptoms^1^. When tested in trials, the use of the diagnoses of poor interpretability, such as frailty, is associated with early termination of trials^32^.

Based on the findings in the present study, several approaches can be used to improve current diagnostic strategies. First, under certain circumstances, single symptoms may achieve high sensitivity or specificity. To effectively detect the disease, single symptoms need to be rare among those not diseased (a low baseline incidence and thus a high specificity) and have high risk ratios of development due to the disease cause (high sensitivity). However, the baseline incidence and risk ratios of the symptoms used to diagnose several conditions, such as frailty^1^ or mental illnesses^2^, have not been well demonstrated.

Second, symptoms should be selected based on evidence, at least on the understanding of possible causes of the symptoms, estimated risk ratios, baseline symptom incidence, and baseline symptom correlations. We noticed that when the risk ratios were similar to 1, the maximal AUCs were around 0.5, and so the AUCs provided little diagnostic values. When the risk ratios were less than 1, suggesting that the presenting symptoms were less likely to be related to the disease, the AUCs were likely to be less than 0.5. When the risk ratios were greater than 1, the AUCs tended to exceed 0.5. When using less than 30 symptoms for disease diagnosis, the AUCs can often reach plateau levels. Epidemiological measures have different impacts on the sensitivities and specificities obtained from the maximal or minimal AUCs using at most 40 symptoms, and assuming risk ratios greater or less than 1, respectively.

Third, when the relationships between symptoms have been well explored, using the number of symptoms for disease diagnosis can effectively minimize the biases introduced by data censoring^1^. The biases induced by data censoring or categorization can lead to a diagnosis, of which more than 70% of its variances can be explained by biases alone^1^.

Fourth, using more symptoms for diagnosis increases complexity. In the present study, when we used more symptoms for diagnosis, we found that their diagnostic accuracy could be improved according to AUCs. However, selecting single symptoms with a high diagnostic accuracy is much preferred because using multiple symptoms requires complex design, depends on well-tested thresholds, and needs to be justified with extensive research on these symptoms and their interactions.

Fifth, baseline symptom correlations are associated with the diagnostic accuracy (AUC) plateau that the symptoms can reach. The differences in the diagnostic accuracy of single symptoms and multiple symptoms are larger when the baseline symptom correlations are closer to 0. It is highly recommended that diagnoses consider the correlations between the symptoms among those diseased or not. Last, in the real world, when the disease cause remains to be investigated, it is not likely to achieve a perfect estimate of baseline symptom incidence or risk ratios, or to confirm baseline symptom correlations among those not diseased. In our simulations, overall symptom correlations, overall symptom incidence, and numbers of symptoms were observable and can be easily obtained. If the risk ratios cannot be estimated at all, symptom correlations and numbers of symptoms are positively and significantly associated with the AUCs for disease diagnosis. The overall symptom incidence is negatively and significantly associated with the AUCs. The three observable measures only explain a small fraction of the variances of the AUCs for disease diagnosis (adjusted R-squared = 0.03). When researchers are confident that these symptoms are more likely to occur among those diseased (RR > 1), these three measures remain significant, although the fraction of the variances of the AUCs for disease diagnosis further decreases (adjusted R-squared = 0.02).

### Future research directions

Several directions are open for future research. First, continuous variables can be used for disease diagnosis, which will require the development of complicated mathematical equations and add complexity to simulation and modeling. We will use the number of symptoms as the template for continuous-variable simulations. Second, often, more than one disease can cause the same symptoms, which adds quite a few interaction terms to the epidemiological measures. When established, these models will provide valuable examples to real-world studies. Third, models that build on incremental improvement will be necessary. It is computationally impossible to implement all models to demonstrate the diagnostic accuracy of the symptoms that occurred based on the epidemiological measures of all possible values. However, it is relatively feasible to construct simulations that conform to well-studied association networks^33,34^ and epidemiological measures reasonably estimated with real-world data. Simulations can be used to support the findings from real world data, and may provide lessons for causal inference. In future studies, we will implement more complicated simulations and explore the usefulness of simulations for causal inference.

Lastly, situations exist that involve more complicated diagnostic approaches, for example clinical case definitions used in outbreak investigations^35,36^. Case definitions may be applicable to patients experiencing symptoms or signs in certain times or places, depending on the diseases of interest^37^. For example, a clinical malaria case can be defined based on the presence of the pathogen in the blood and the occurrence of related symptoms within 2 days of examination^38^. These case definitions can be modified to suit outbreak investigations and settings^39^. Our findings help to demonstrate the key epidemiological parameters that researchers need to pay attention to when they aim to update case definitions. In an outbreak investigation, the information on these epidemiological measures should be systematically collected. We think it possible to improve case definitions using updated information on these measures. This finding needs to be studied further in the future.

### Limitations

Our simulation study depended on various assumptions: one disease causing multiple symptoms, similar symptom incidence, similar risk ratios causing symptoms, and similar correlations between symptoms among those not diseased. A related disease was set up to occur in association with the symptom-causing disease. This related disease remains insignificant in the symptoms’ diagnostic accuracy for disease diagnosis (AUCs, sensitivities, and specificities). However, the simulations are not likely to match the complex multi-cause examples commonly seen in the real world. For example, the symptoms of frailty, a geriatric syndrome, can be linked to frailty and many other causes^1,6^. Due to computational constraints, a limited number of the values of the epidemiological measures were simulated. These epidemiological measures have many other values that need to be tested. Assuming the epidemiological measures have similar levels across symptoms, variations are due to the random assignment to different simulated populations. These variations may lead to slight differences in the simulation results.

## Conclusion

Assuming symptoms are caused by a single disease, they occur based on four epidemiological measures: proportions diseased, baseline symptom incidence, risk ratios, and baseline symptom correlations. The symptom incidence among those diseased, at-risk incidence, can reach a maximum of 1. The sensitivities and specificities of single symptoms for disease diagnosis can be fully predicted by at-risk incidence and 1 minus baseline incidence, respectively. When the disease causes multiple symptoms based on similar epidemiological measures, these symptoms can be used for disease diagnosis. Using two symptoms for disease diagnosis—for example, the sensitivities and specificities of having 0, 1, or 2 symptoms—can be calculated to draw a ROC and derive its AUC. When repeating the same procedures using 1 to 40 symptoms for disease diagnosis, the maximal AUCs can be obtained, and the best sets of sensitivities and specificities can be selected from them. The above-mentioned epidemiological measures can explain large fractions of the maximal AUCs and the best sets of sensitivities and specificities. These findings are important for researchers who want to assess composite diagnostic criteria that are subject to biases and lack an evidence base. For example, the recommendations on constructing a frailty index have been widely used^7^. However, these recommendations neglect the role of these epidemiological measures and focus on observable measures (overall symptom incidence and numbers of symptoms) that do not well explain symptom diagnostic accuracy.

## Supplementary Information

Supplementary Information.

## References

[1] Chao Y-S, Wu H-C, Wu C-J, Chen W-C (2018). Index or illusion: The case of frailty indices in the Health and Retirement Study. PLoS ONE.

[2] Chao Y-S, Lin K-F, Wu C-J, Wu H-C, Hsu H-T, Tsao L-C (2020). Simulation study to demonstrate biases created by diagnostic criteria of mental illnesses: Major depressive episodes, dysthymia, and manic episodes. BMJ Open.

[3] Soares-Weiser K, Maayan N, Bergman H, Davenport C, Kirkham AJ, Grabowski S (2015). First rank symptoms for schizophrenia (Cochrane diagnostic test accuracy review). Schizophr. Bull..

[4] American Psychiatric Association (2010). Diagnostic and Statistical Manual of Mental Disorders, Fourth Edition, Text Revision (DSM-IV-TR®).

[5] Chao Y-S, McGolrick D, Wu C-J, Wu H-C, Chen W-C (2019). A proposal for a self-rated frailty index and status for patient-oriented research. BMC Res. Notes.

[6] Cigolle CT, Ofstedal MB, Tian Z, Blaum CS (2009). Comparing models of frailty: The Health and Retirement Study. J. Am. Geriatr. Soc..

[7] Searle SD, Mitnitski A, Gahbauer EA, Gill TM, Rockwood K (2008). A standard procedure for creating a frailty index. BMC Geriatr..

[8] Chao Y-S, Wu C-J, Wu H-C, Hsu H-T, Tsao L-C, Cheng Y-P (2020). Using syndrome mining with the Health and Retirement Study to identify the deadliest and least deadly frailty syndromes. Sci. Rep..

[9] Chao Y-S, Wu C-J, Wu H-C, Hsu H-T, Tsao L-C, Cheng Y-P (2020). Composite diagnostic criteria are problematic for linking potentially distinct populations: The case of frailty. Sci. Rep..

[10] Vetrano DL, Palmer K, Marengoni A, Marzetti E, Lattanzio F, Roller-Wirnsberger R (2018). Frailty and multimorbidity: A systematic review and meta-analysis. J. Gerontol. Ser. A.

[11] Baratloo, A., Hosseini, M., Negida, A. & El Ashal, G. Part 1: Simple definition and calculation of accuracy, sensitivity and specificity (2015). PMC4614595 26495380

[12] Gordts F, Clement PAR, Destryker A, Desprechins B, Kaufman L (1997). Prevalence of sinusitis signs on MRI in a non-ENT paediatric population. Rhinology.

[13] Smatti MK, Al-Sadeq DW, Ali NH, Pintus G, Abou-Saleh H, Nasrallah GK (2018). Epstein–Barr virus epidemiology, serology, and genetic variability of LMP-1 oncogene among healthy population: an update. Front. Oncol..

[14] Weiss H (2004). Epidemiology of herpes simplex virus type 2 infection in the developing world. Herpes J. IHMF.

[15] Davies AR, Ruggles R, Young Y, Clark H, Reddell P, Verlander NQ (2013). *Salmonella enterica* serovar Enteritidis phage type 4 outbreak associated with eggs in a large prison, London 2009: An investigation using cohort and case/non-case study methodology. Epidemiol. Infect..

[16] Shun CB, Donaghue KC, Phelan H, Twigg SM, Craig ME (2014). Thyroid autoimmunity in Type 1 diabetes: Systematic review and meta-analysis. Diabet. Med..

[17] Arscott-Mills S (2001). Intimate partner violence in Jamaica: A descriptive study of women who access the services of the Women's Crisis Centre in Kingston. Violence Against Women.

[18] Leisch, F., Weingessel, A. & Hornik, K. On the generation of correlated artificial binary data (1998).

[19] Leisch F, Weingessel A, Leisch MF (2006). The Bindata Package.

[20] Chao, Y. S. *et al.**HPV Testing for Primary Cervical Cancer Screening: A Health Technology Assessment* (Canadian Agency for Drugs and Technologies in Health, 2019). Available from https://www.cadth.ca/sites/default/files/ou-tr/op0530-hpv-testing-for-pcc-report.pdf. 31246380

[21] Robin X, Turck N, Hainard A, Tiberti N, Lisacek F, Sanchez J-C (2011). pROC: An open-source package for R and S+ to analyze and compare ROC curves. BMC Bioinform..

[22] Ray P, Le Manach Y, Riou B, Houle TT (2010). Statistical evaluation of a biomarker. Anesthesiol. J. Am. Soc. Anesthesiol..

[23] Chao Y-S, Wu C-J (2019). PD25 principal component approximation: Medical expenditure panel survey. Int. J. Technol. Assess. Health Care.

[24] Chao Y-S, Wu H-C, Wu C-J, Chen W-C (2018). Principal component approximation and interpretation in Health Survey and Biobank Data. Front. Digit. Humanit..

[25] Chao Y-S, Wu C-J (2017). Principal component-based weighted indices and a framework to evaluate indices: Results from the Medical Expenditure Panel Survey 1996 to 2011. PLoS ONE.

[26] Chao YS, Wu HC, Wu CJ, Chen WC (2018). Stages of biological development across age: An analysis of Canadian Health Measure Survey 2007–2011. Front. Public Health.

[27] Chao YS, Wu HT, Wu CJ (2017). Feasibility of classifying life stages and searching for the determinants: Results from the Medical Expenditure Panel Survey 1996–2011. Front. Public Health.

[28] R Development Core Team (2016). R: A Language and Environment for Statistical Computing.

[29] RStudio Team (2016). RStudio: Integrated Development for R.

[30] Chao Y-S, Wu C-J, Lai Y-C, Hsu H-T, Cheng Y-P, Wu H-C (2022). Why mental illness diagnoses are wrong: A pilot study on the perspectives of the public. Front. Psychiatry.

[31] Chao Y-S, Wu C-J (2019). PP46 when composite measures or indices fail: Data processing lessons. Int. J. Technol. Assess. Health Care.

[32] Chao Y-S, Wu C-J, Wu H-C, McGolrick D, Chen W-C (2021). Interpretable trials: Is interpretability a reason why clinical trials fail?. Front. Med..

[33] Chao Y-S, Scutari M, Chen T-S, Wu C-J, Durand M, Boivin A (2018). A network perspective of engaging patients in specialist and chronic illness care: The 2014 International Health Policy Survey. PLoS ONE.

[34] Chao YS, Wu HT, Scutari M, Chen TS, Wu CJ, Durand M (2017). A network perspective on patient experiences and health status: The Medical Expenditure Panel Survey 2004 to 2011. BMC Health Serv. Res..

[35] Keou FX, Belec L, Esunge PM, Cancre N, Gresenguet G (1992). World Health Organization clinical case definition for AIDS in Africa: An analysis of evaluations. East Afr. Med. J..

[36] Pharagood-Wade, F., Swirsky, L. & Teran-MacIver, M. Disease clusters; an overview.

[37] Collin L, Reisner SL, Tangpricha V, Goodman M (2016). Prevalence of transgender depends on the “case” definition: A systematic review. J. Sex. Med..

[38] Afrane YA, Zhou G, Githeko AK, Yan G (2014). Clinical malaria case definition and malaria attributable fraction in the highlands of western Kenya. Malar. J..

[39] Bosman, A. *Case Definitions for Outbreak Assessment* (European Centre for Disease Prevention and Control (ECDC), 2012). Available from https://wiki.ecdc.europa.eu/fem/Pages/Case%20definitions%20for%20outbreak%20assessment.aspx.

