# Supplementary material for: Diagnostic accuracy of symptoms for an underlying disease: a simulation study
Source: Sci Rep. 2022 Aug 15;12:13810. doi: 10.1038/s41598-022-14826-2 (PMC9378763; doi:10.1038/s41598-022-14826-2)
Supplement: Supplementary file 1 — Supplementary Information. [file 41598_2022_14826_MOESM1_ESM.docx]

Diagnostic accuracy of symptoms for an underlying disease: a simulation study

# Authors

Yi-Sheng Chao, Chao-Jung Wu, Yi-Chun Lai, Hui-Ting Hsu, Yen-Po Cheng, Hsing-Chien Wu, Shih-Yu Huang, Wei-Chih Chen

# Appendix 1

Copy the R codes below to a RMD file.

---

title: "2020_02_29 different RR to symptom cor"

author: "Yi-Sheng Chao"

date: "Febuary 20, 2020"

output: pdf_document

---

The DTA gradient from high to low relative risks

```{r}

options(warn=-1)

#set up a single variable

library(pROC)

library(ggplot2)

library(rsq)

library(caret)

library(bindata)

library(Hmisc)

# pROC_obj$auc

# pROC_obj$ci[1]

memory.limit(size = 10^13)

ssize = 10^4

times = 10^1

nvar = 40

# prop = c(0.05, 0.1, 0.3, 0.5, 0.7)

# dxcor = c(0, 0.05, 0.1, 0.3, 0.5, 0.7, 0.9)#correlations between the true causes of MDE, DYS, and Manic

dxcor = c(0, 0.3, 0.7)#correlations between the true causes of MDE, DYS, and Manic

dxrisk = c(0.05, 0.1, 0.2, 0.4, 0.8)#proportions of populations at risk

dxpreval = c(0.05, 0.1, 0.2, 0.4, 0.8)#inciddence of dx in populations not at risk

relativer = c(0.5, 1, 2, 10, 25)#relative risks of developing dx in at-riks populations

symcor = c(0, 0.4, 0.8)#correlations between the symptoms

symcordi = c(1)

collect = unique(c("mean", "max", "min", "overlapmean", "derivedrisk", "derivedcor", "derivedsymcor", "derivedir","derivedbasesymcor", "derivedbasesymcor_p", "derivedrisksymcor", "derivedrisksymcor_p", "derivedrr", "derivedrisk_dys", "derivedcor_dys", "derivedir_dys", "derivedrr_dys", "derivedirrr_dys", "obsrr", "derivedirrr","derivedprevalence", "derivedir_dys", "derivedrr_dys", "derivedir_man", "derivedrr_man", "mdepreval", "dyspreval", "manicpreval", "cor_mde", "cor_dys", "cor_man", "cor_sym", "cor_p_mde", "cor_p_dys", "cor_p_man", "cor_p_sym", "chi_mde", "chi_dys", "chi_man", "chi_sym", "chi_p_mde", "chi_p_dys", "chi_p_man", "chi_p_sym", "sen_mde", "spe_mde", "ppv_mde", "npv_mde", "sen_dys", "spe_dys", "ppv_dys", "npv_dys", "sen_man", "spe_man", "ppv_man", "npv_man", "sen_mde_max", "sen_mde_maxn", "spe_mde_max", "spe_mde_maxn", "sen_mde_mdm", "spe_mde_mdm", "sen_mde_min", "spe_mde_min", "auc_mde", "auc_mde_n", "auc_mde_95up", "auc_mde_95lo", "auc_mde", "auc_mde_n", "auc_mde_95up", "auc_mde_95lo", "sen_dys_max", "spe_dys_max", "auc_dys", "auc_dys_n", "auc_dys_95up", "auc_dys_95lo", "sen_man_max", "spe_man_max", "auc_man", "auc_man_n", "auc_man_95up", "auc_man_95lo", "derivedrr", "reg_mde", "reg_mde_95up", "reg_mde_95lo", "reg_mde_se", "reg_p_mde", "reg_p_mde_95up", "reg_p_mde_95lo", "reg_p_mde_se", "reg_r2_mde", "reg_r2_mde_95up", "reg_r2_mde_95lo", "reg_r2_mde_se", "reg_int_mde", "reg_int_mde_95up", "reg_int_mde_95lo", "reg_int_mde_se", "reg_intp_mde", "reg_intp_mde_95up", "reg_intp_mde_95lo", "reg_intp_mde_se", "reg_dys", "reg_dys_95up", "reg_dys_95lo", "reg_dys_se", "reg_p_dys", "reg_p_dys_95up", "reg_p_dys_95lo", "reg_p_dys_se", "reg_r2_dys", "reg_r2_dys_95up", "reg_r2_dys_95lo", "reg_r2_dys_se", "reg_int_dys", "reg_int_dys_95up", "reg_int_dys_95lo", "reg_int_dys_se", "reg_intp_dys", "reg_intp_dys_95up", "reg_intp_dys_95lo", "reg_intp_dys_se", "reg_sym", "reg_sym_95up", "reg_sym_95lo", "reg_sym_se", "reg_p_sym", "reg_p_sym_95up", "reg_p_sym_95lo", "reg_p_sym_se", "reg_r2_sym", "reg_r2_sym_95up", "reg_r2_sym_95lo", "reg_r2_sym_se", "reg_int_sym", "reg_int_sym_95up", "reg_int_sym_95lo", "reg_int_sym_se", "reg_intp_sym", "reg_intp_sym_95up", "reg_intp_sym_95lo", "reg_intp_sym_se", "reg_mdeboth", "reg_mdeboth_95up", "reg_mdeboth_95lo", "reg_mdeboth_se", "reg_dysboth", "reg_dysboth_95up", "reg_dysboth_95lo", "reg_dysboth_se", "reg_p_mdeboth", "reg_p_mdeboth_95up", "reg_p_mdeboth_95lo", "reg_p_mdeboth_se", "reg_p_dysboth", "reg_p_dysboth_95up", "reg_p_dysboth_95lo", "reg_p_dysboth_se", "reg_r2_both", "reg_r2_both_95up", "reg_r2_both_95lo", "reg_r2_both_se", "reg_int_both", "reg_int_both_95up", "reg_int_both_95lo", "reg_int_both_se", "reg_intp_both", "reg_intp_both_95up", "reg_intp_both_95lo", "reg_intp_both_se" ))

collect = unique(collect)

checkass = data.frame(matrix(c("Assumed correlations\nbetween true causes", "Correlations\nbetween true diseases", "Assumed proportions at risk", "Proportions at risk", "Assumed relative risk", "Relative risk", "Assumed disease incidence", "Incidence of pop not at risk", "Assumed incidence\nof input symptoms (dxpreval)", "Major depressive episodes", "Dysthymic disorder", "Manic episodes"), nrow = 1))

i = nrow(checkass)

set.seed(1)

for(dc in 1:length(dxcor)){

for(dr in 1:length(dxrisk)){

for(rr in 1:length(relativer)){

for(dp in 1:length(dxpreval)){

for(sc in 1:length(symcor)){

for(scd in 1:length(symcordi)){

#if the incidence too low the correlation matrix won't work

if((as.numeric(relativer[rr]) * as.numeric(dxpreval[dp])) > 0.1){

#Incidence rates should be less than or equal to 1.0

# if((as.numeric(relativer[rr]) * as.numeric(dxpreval[dp]))<=1){

# for(ass in assum){

# library(openxlsx)

resu = data.frame(matrix(c("female", "age", "edu", paste("X", 1:nvar, sep = "")), ncol = 1))#load data for

names(resu) = "variable"

for(t in 1:times){

##demographic characteristics independently generated

sim = NA

sim = data.frame(1:ssize)

names(sim) = "id"

sim$female = rbinom(n = ssize, size = 1, prob = 0.51)

sim$age = sample(30:60, ssize, replace = TRUE)

sim$edu = rnorm(ssize, mean = 12, sd = 5)

sim$edu[which(sim$edu <= 0)] = 0

sim$id = NULL

# library(bindata)

tempbin = as.data.frame(rmvbin(ssize, rep(dxrisk[dr], 4), bincorr=(1 - dxcor[dc])*diag(4) + dxcor[dc]))

sim$mdecause = tempbin[,1]

# sim$mdecause[1:(ssize*dxrisk[dr])] = 1

sim$maniccause = tempbin[,2]

# sim$maniccause[which(sim$mdecause == 0)] = c(rep(1, ssize*dxrisk[dr]), rep(0, (ssize * (1- 2*prop))))

sim$dyscause = tempbin[,3]

# if(assum[ass] == 2){

# sim$dyscause = sim$mdecause

# }

sim[,paste("X", 1:nvar, sep = "")] = NA

# bindata = data.frame(matrix(rep(NA, (ssize*nvar)), nrow = ssize))

# bindata2 = data.frame(matrix(rep(NA, (ssize*nvar)), nrow = ssize))

# dim(bindata)

# for(c in 1:nvar){

###the incidence of symptoms is top-censored at 100%

if((as.numeric(relativer[rr]) * as.numeric(dxpreval[dp])) < 1){

sim[which(sim$mdecause == 1), paste("X", 1:nvar, sep = "")] = as.data.frame(rmvbin(nrow(sim[which(sim$mdecause == 1), ]), rep((as.numeric(relativer[rr]) * as.numeric(dxpreval[dp])), nvar), bincorr=(1 - symcor[sc])*diag(nvar) + symcor[sc]))

}

if((as.numeric(relativer[rr]) * as.numeric(dxpreval[dp])) >= 1){

sim[which(sim$mdecause == 1), paste("X", 1:nvar, sep = "")] = 1

}

sim[which(sim$mdecause == 0), paste("X", 1:nvar, sep = "")] = as.data.frame(rmvbin(nrow(sim[which(sim$mdecause == 0), ]), rep((1 * as.numeric(dxpreval[dp])), nvar), bincorr=(1 - symcor[sc]*symcordi[scd])*diag(nvar) + symcor[sc]*symcordi[scd]))

#End of generating variables

#Varaible absolute values for diagnostic test accuracy

for(simc in 1:ncol(sim)){

if(any(sim[,simc] <0)){sim[,simc] = abs(sim[,simc])}

}

#end of variable absolute values

#Diagnosis interpretation

resu[, paste(collect, "_", t, sep = "")] = NA

resu$variable = as.character(resu$variable)

for(r in 1:nrow(resu)){

#variable characteristics

if(is.na(resu$variable[r]) == FALSE){

# "derivedrisk", "derivedcor", "derivedsymcor"

resu[r, paste0("derivedrisk_", t, collapse = "")] = nrow(sim[which(sim$mdecause == 1),])/ssize

resu[r, paste0("derivedcor_", t, collapse = "")] = cor(sim$mdecause, sim$maniccause)

if(r<nrow(resu)){

resu[r, paste0("derivedsymcor_", t, collapse = "")] = cor(sim[, as.character(resu$variable[r])], sim[,as.character(resu$variable[r+1])])

resu[r, paste0("derivedsymcor_p_", t, collapse = "")] = cor.test(sim[, as.character(resu$variable[r])], sim[,as.character(resu$variable[r+1])])$p.value

resu[r, paste0("derivedbasesymcor_", t, collapse = "")] = cor(sim[which(sim$mdecause == 0), as.character(resu$variable[r])], sim[which(sim$mdecause == 0),as.character(resu$variable[r+1])])

resu[r, paste0("derivedbasesymcor_p_", t, collapse = "")] = cor.test(sim[which(sim$mdecause == 0), as.character(resu$variable[r])], sim[which(sim$mdecause == 0),as.character(resu$variable[r+1])])$p.value

resu[r, paste0("derivedrisksymcor_", t, collapse = "")] = cor(sim[which(sim$mdecause == 1), as.character(resu$variable[r])], sim[which(sim$mdecause == 1),as.character(resu$variable[r+1])])

resu[r, paste0("derivedrisksymcor_p_", t, collapse = "")] = cor.test(sim[which(sim$mdecause == 1), as.character(resu$variable[r])], sim[which(sim$mdecause == 1),as.character(resu$variable[r+1])])$p.value

}

if(r==nrow(resu)){

resu[r, paste0("derivedsymcor_", t, collapse = "")] = cor(sim[, as.character(resu$variable[r])], sim[,as.character(resu$variable[r-1])])

resu[r, paste0("derivedsymcor_p_", t, collapse = "")] = cor.test(sim[, as.character(resu$variable[r])], sim[,as.character(resu$variable[r-1])])$p.value

resu[r, paste0("derivedbasesymcor_", t, collapse = "")] = cor(sim[which(sim$mdecause == 0), as.character(resu$variable[r])], sim[which(sim$mdecause == 0),as.character(resu$variable[r-1])])

resu[r, paste0("derivedbasesymcor_p_", t, collapse = "")] = cor.test(sim[which(sim$mdecause == 0), as.character(resu$variable[r])], sim[which(sim$mdecause == 0),as.character(resu$variable[r-1])])$p.value

resu[r, paste0("derivedrisksymcor_", t, collapse = "")] = cor(sim[which(sim$mdecause == 1), as.character(resu$variable[r])], sim[which(sim$mdecause == 1),as.character(resu$variable[r-1])])

resu[r, paste0("derivedrisksymcor_p_", t, collapse = "")] = cor.test(sim[which(sim$mdecause == 1), as.character(resu$variable[r])], sim[which(sim$mdecause == 1),as.character(resu$variable[r-1])])$p.value

}

resu[r, paste0("derivedprevalence_", t, collapse = "")] = nrow(sim[which(sim[, as.character(resu$variable[r])] == 1),])/ssize

#"derivedrisk_dys", "derivedcor_dys", "derivedir_dys", "derivedrr_dys"

resu[r, paste0("derivedrisk_cor_", t, collapse = "")] = nrow(sim[which(sim$dyscause == 1),])/ssize

resu[r, paste0("derivedir_dys_", t, collapse = "")] = (nrow(sim[which(sim$dyscause == 0 & sim[, as.character(resu$variable[r])] == 1),])/nrow(sim[which(sim$dyscause == 0),]))

resu[r, paste0("derivedrr_dys_", t, collapse = "")] = (nrow(sim[which(sim$dyscause == 1 & sim[, as.character(resu$variable[r])] == 1),])/nrow(sim[which(sim$dyscause == 1),]))/(nrow(sim[which(sim$dyscause == 0 & sim[,as.character(resu$variable[r])] == 1),])/nrow(sim[which(sim$dyscause == 0),]))

resu[r, paste0("derivedirrr_dys_", t, collapse = "")] = (nrow(sim[which(sim$dyscause == 1 & sim[, as.character(resu$variable[r])] == 1),])/nrow(sim[which(sim$dyscause == 1),]))

resu[r, paste0("mean_", t, collapse = "")] = mean(sim[,as.character(resu$variable[r])])

resu[r, paste0("max_", t, collapse = "")] = max(sim[, as.character(resu$variable[r])])

resu[r, paste0("min_", t, collapse = "")] = min(sim[, as.character(resu$variable[r])])

resu[r, paste0("mdepreval_", t, collapse = "")] = nrow(sim[which(sim$mdecause == 1),])/nrow(sim)

resu[r, paste0("dyspreval_", t, collapse = "")] = nrow(sim[which(sim$dyscause == 1),])/nrow(sim)

resu[r, paste0("manicpreval_", t, collapse = "")] = nrow(sim[which(sim$maniccause == 1),])/nrow(sim)

# library(Hmisc)

if(as.character(resu$variable[r]) %in% paste("X", 1:nvar, sep = "")){

resu[r, paste0("overlapmean_", t, collapse = "")] = nrow(sim[which(sim$X1 == 1), ])

if(as.character(resu$variable[r]) %in% paste("X", 2:nvar, sep = "")){resu[r, paste0("overlapmean_", t, collapse = "")] = nrow(sim[which(rowSums(sim[,paste("X", 1:as.numeric(gsub("X", "", as.character(resu$variable[r]))), sep = "")]) == as.numeric(gsub("X", "", as.character(resu$variable[r])))), ])}

resu[r, paste0("derivedir_", t, collapse = "")] = (nrow(sim[which(sim$mdecause == 0 & sim[, as.character(resu$variable[r])] == 1),])/nrow(sim[which(sim$mdecause == 0),]))

resu[r, paste0("derivedrr_", t, collapse = "")] = (nrow(sim[which(sim$mdecause == 1 & sim[, as.character(resu$variable[r])] == 1),])/nrow(sim[which(sim$mdecause == 1),]))/(nrow(sim[which(sim$mdecause == 0 & sim[,as.character(resu$variable[r])] == 1),])/nrow(sim[which(sim$mdecause == 0),]))

resu[r, paste0("derivedirrr_", t, collapse = "")] = (nrow(sim[which(sim$mdecause == 1 & sim[, as.character(resu$variable[r])] == 1),])/nrow(sim[which(sim$mdecause == 1),]))

resu[r, paste0("derivedir_dys_", t, collapse = "")] = (nrow(sim[which(sim$dyscause == 0 & sim[, as.character(resu$variable[r])] == 1),])/nrow(sim[which(sim$dyscause == 0),]))

resu[r, paste0("derivedrr_dys_", t, collapse = "")] = (nrow(sim[which(sim$dyscause == 1 & sim[, as.character(resu$variable[r])] == 1),])/nrow(sim[which(sim$dyscause == 1),]))/(nrow(sim[which(sim$dyscause == 0 & sim[,as.character(resu$variable[r])] == 1),])/nrow(sim[which(sim$dyscause == 0),]))

resu[r, paste0("derivedir_man_", t, collapse = "")] = (nrow(sim[which(sim$maniccause == 0 & sim[, as.character(resu$variable[r])] == 1),])/nrow(sim[which(sim$maniccause == 0),]))

resu[r, paste0("derivedrr_man_", t, collapse = "")] = (nrow(sim[which(sim$maniccause == 1 & sim[, as.character(resu$variable[r])] == 1),])/nrow(sim[which(sim$maniccause == 1),]))/(nrow(sim[which(sim$maniccause == 0 & sim[,as.character(resu$variable[r])] == 1),])/nrow(sim[which(sim$maniccause == 0),]))

resu[r, paste0("obsrr_", t, collapse = "")] = nrow(sim[which(sim[, as.character(resu$variable[r])] == 1),])/nrow(sim[which(sim[, as.character(resu$variable[r])] == 0),])

resu[r, paste0("cor_mde_", t, collapse = "")] = cor(sim[, as.character(resu$variable[r])],sim$mdecause)

resu[r, paste0("cor_dys_", t, collapse = "")] = cor(sim[, as.character(resu$variable[r])],sim$dyscause)

resu[r, paste0("cor_man_", t, collapse = "")] = cor(sim[, as.character(resu$variable[r])],sim$maniccause)

resu[r, paste0("cor_p_mde_", t, collapse = "")] = rcorr(sim[, as.character(resu$variable[r])],sim$mdecause)$P[2]

resu[r, paste0("cor_p_dys_", t, collapse = "")] = rcorr(sim[, as.character(resu$variable[r])],sim$dyscause)$P[2]

resu[r, paste0("cor_p_man_", t, collapse = "")] = rcorr(sim[, as.character(resu$variable[r])],sim$maniccause)$P[2]

if(as.character(resu$variable[r]) %in% paste("X", 1:(nvar-1), sep = "")){

resu[r, paste0("cor_sym_", t, collapse = "")] = cor(sim[, as.character(resu$variable[r])],sim[, as.character(resu$variable[r+1])])

resu[r, paste0("cor_p_sym_", t, collapse = "")] = rcorr(sim[, as.character(resu$variable[r])],sim[, as.character(resu$variable[r+1])])$P[2]}#if not the last variable

if(as.character(resu$variable[r]) == paste("X", nvar, sep = "")){

resu[r, paste0("cor_sym_", t, collapse = "")] = cor(sim[, as.character(resu$variable[r])],sim$X1)

resu[r, paste0("cor_sym_", t, collapse = "")] = rcorr(sim[, as.character(resu$variable[r])],sim$X1)$P[2]

}#if resu$variable is the last variable

resu[r, paste0("chi_mde_", t, collapse = "")] = chisq.test(sim[, as.character(resu$variable[r])],sim$mdecause)$statistic

resu[r, paste0("chi_dys_", t, collapse = "")] = chisq.test(sim[, as.character(resu$variable[r])],sim$dyscause)$statistic

resu[r, paste0("chi_man_", t, collapse = "")] = chisq.test(sim[, as.character(resu$variable[r])],sim$maniccause)$statistic

resu[r, paste0("chi_p_mde_", t, collapse = "")] = chisq.test(sim[, as.character(resu$variable[r])],sim$mdecause)$p.value

resu[r, paste0("chi_p_dys_", t, collapse = "")] = chisq.test(sim[, as.character(resu$variable[r])],sim$dyscause)$p.value

resu[r, paste0("chi_p_man_", t, collapse = "")] = chisq.test(sim[, as.character(resu$variable[r])],sim$maniccause)$p.value

if(as.character(resu$variable[r]) %in% paste("X", 1:(nvar-1), sep = "")){

resu[r, paste0("chi_sym_", t, collapse = "")] = chisq.test(sim[, as.character(resu$variable[r])],sim[, as.character(resu$variable[r+1])])$statistic

resu[r, paste0("chi_p_sym_", t, collapse = "")] = chisq.test(sim[, as.character(resu$variable[r])],sim[, as.character(resu$variable[r+1])])$p.value}#if not the last variable

if(as.character(resu$variable[r]) == paste("X", nvar, sep = "")){

resu[r, paste0("chi_sym_", t, collapse = "")] = chisq.test(sim[, as.character(resu$variable[r])],sim$X1)$statistic

resu[r, paste0("chi_p_sym_", t, collapse = "")] = chisq.test(sim[, as.character(resu$variable[r])],sim$X1)$p.value

}#if resu$variable is the last variable

#symptom interpretation by the disease using linear regression

##reg: variable = MDE * beta

##rereg: reverse MDE = variable* beta

#interpretation with the disease

templm = summary(lm(as.formula(paste0(resu$variable[r], "~mdecause", collapse = "")),sim))

resu[r, paste0("reg_mde_", t, collapse = "")] = templm$coefficients["mdecause", "Estimate"]

resu[r, paste0("reg_p_mde_", t, collapse = "")] = templm$coefficients["mdecause", "Pr(>|t|)"]

resu[r, paste0("reg_int_mde_", t, collapse = "")] = templm$coefficients["(Intercept)", "Estimate"]

resu[r, paste0("reg_intp_mde_", t, collapse = "")] = templm$coefficients["(Intercept)", "Pr(>|t|)"]

resu[r, paste0("reg_r2_mde_", t, collapse = "")] = templm$adj.r.squared

#interpretatiojn with the other related disease

templm = summary(lm(as.formula(paste0(resu$variable[r], "~dyscause", collapse = "")),sim))

resu[r, paste0("reg_dys_", t, collapse = "")] = templm$coefficients["dyscause", "Estimate"]

resu[r, paste0("reg_p_dys_", t, collapse = "")] = templm$coefficients["dyscause", "Pr(>|t|)"]

resu[r, paste0("reg_int_dys_", t, collapse = "")] = templm$coefficients["(Intercept)", "Estimate"]

resu[r, paste0("reg_intp_dys_", t, collapse = "")] = templm$coefficients["(Intercept)", "Pr(>|t|)"]

resu[r, paste0("reg_r2_dys_", t, collapse = "")] = templm$adj.r.squared

#interpretatiojn with the disease and the other related disease

templm = summary(lm(as.formula(paste0(resu$variable[r], "~mdecause+dyscause", collapse = "")),sim))

resu[r, paste0("reg_mdeboth_", t, collapse = "")] = templm$coefficients["mdecause", "Estimate"]

resu[r, paste0("reg_dysboth_", t, collapse = "")] = templm$coefficients["dyscause", "Estimate"]

resu[r, paste0("reg_p_mdeboth_", t, collapse = "")] = templm$coefficients["mdecause", "Pr(>|t|)"]

resu[r, paste0("reg_p_dysboth_", t, collapse = "")] = templm$coefficients["dyscause", "Pr(>|t|)"]

resu[r, paste0("reg_int_both_", t, collapse = "")] = templm$coefficients["(Intercept)", "Estimate"]

resu[r, paste0("reg_intp_both_", t, collapse = "")] = templm$coefficients["(Intercept)", "Pr(>|t|)"]

resu[r, paste0("reg_r2_both_", t, collapse = "")] = templm$adj.r.squared

#interpretatiojn with the disease, another and the other related disease

templm = summary(lm(as.formula(paste0(resu$variable[r], "~mdecause+dyscause+maniccause", collapse = "")),sim))

resu[r, paste0("reg_mdeall_", t, collapse = "")] = templm$coefficients["mdecause", "Estimate"]

resu[r, paste0("reg_dysall_", t, collapse = "")] = templm$coefficients["dyscause", "Estimate"]

resu[r, paste0("reg_manall_", t, collapse = "")] = templm$coefficients["maniccause", "Estimate"]

resu[r, paste0("reg_p_mdeall_", t, collapse = "")] = templm$coefficients["mdecause", "Pr(>|t|)"]

resu[r, paste0("reg_p_dysall_", t, collapse = "")] = templm$coefficients["dyscause", "Pr(>|t|)"]

resu[r, paste0("reg_p_manall_", t, collapse = "")] = templm$coefficients["maniccause", "Pr(>|t|)"]

resu[r, paste0("reg_int_all_", t, collapse = "")] = templm$coefficients["(Intercept)", "Estimate"]

resu[r, paste0("reg_intp_all_", t, collapse = "")] = templm$coefficients["(Intercept)", "Pr(>|t|)"]

resu[r, paste0("reg_r2_all_", t, collapse = "")] = templm$adj.r.squared

#interpretatiojn with the other symptoms

if(as.character(resu$variable[r]) != "X1"){

templm = summary(lm(as.formula(paste0(resu$variable[r], "~X1", collapse = "")),sim))

resu[r, paste0("reg_sym_", t, collapse = "")] = templm$coefficients["X1", "Estimate"]

resu[r, paste0("reg_p_sym_", t, collapse = "")] = templm$coefficients["X1", "Pr(>|t|)"]

resu[r, paste0("reg_int_sym_", t, collapse = "")] = templm$coefficients["(Intercept)", "Estimate"]

resu[r, paste0("reg_intp_sym_", t, collapse = "")] = templm$coefficients["(Intercept)", "Pr(>|t|)"]

resu[r, paste0("reg_r2_sym_", t, collapse = "")] = templm$adj.r.squared

}#Not X1

if(as.character(resu$variable[r]) == "X1"){

templm = summary(lm(as.formula(paste0(resu$variable[r], "~X2", collapse = "")),sim))

resu[r, paste0("reg_sym_", t, collapse = "")] = templm$coefficients["X2", "Estimate"]

resu[r, paste0("reg_p_sym_", t, collapse = "")] = templm$coefficients["X2", "Pr(>|t|)"]

resu[r, paste0("reg_int_sym_", t, collapse = "")] = templm$coefficients["(Intercept)", "Estimate"]

resu[r, paste0("reg_intp_sym_", t, collapse = "")] = templm$coefficients["(Intercept)", "Pr(>|t|)"]

resu[r, paste0("reg_r2_sym_", t, collapse = "")] = templm$adj.r.squared

}#For X1 only

#interpretatiojn with the other symptoms and mdecause

if(as.character(resu$variable[r]) != "X1"){

templm = summary(lm(as.formula(paste0(resu$variable[r], "~X1+mdecause", collapse = "")),sim))

resu[r, paste0("reg_symmde_", t, collapse = "")] = templm$coefficients["X1", "Estimate"]

resu[r, paste0("reg_p_symmde_", t, collapse = "")] = templm$coefficients["X1", "Pr(>|t|)"]

resu[r, paste0("reg_mdemde_", t, collapse = "")] = templm$coefficients["mdecause", "Estimate"]

resu[r, paste0("reg_p_mdemde_", t, collapse = "")] = templm$coefficients["mdecause", "Pr(>|t|)"]

resu[r, paste0("reg_int_symmde_", t, collapse = "")] = templm$coefficients["(Intercept)", "Estimate"]

resu[r, paste0("reg_intp_symmde_", t, collapse = "")] = templm$coefficients["(Intercept)", "Pr(>|t|)"]

resu[r, paste0("reg_r2_symmde_", t, collapse = "")] = templm$adj.r.squared

}#Not X1

if(as.character(resu$variable[r]) == "X1"){

templm = summary(lm(as.formula(paste0(resu$variable[r], "~X2+mdecause", collapse = "")),sim))

resu[r, paste0("reg_symmde_", t, collapse = "")] = templm$coefficients["X2", "Estimate"]

resu[r, paste0("reg_p_symmde_", t, collapse = "")] = templm$coefficients["X2", "Pr(>|t|)"]

resu[r, paste0("reg_mdemde_", t, collapse = "")] = templm$coefficients["mdecause", "Estimate"]

resu[r, paste0("reg_p_mdemde_", t, collapse = "")] = templm$coefficients["mdecause", "Pr(>|t|)"]

resu[r, paste0("reg_int_symmde_", t, collapse = "")] = templm$coefficients["(Intercept)", "Estimate"]

resu[r, paste0("reg_intp_symmde_", t, collapse = "")] = templm$coefficients["(Intercept)", "Pr(>|t|)"]

resu[r, paste0("reg_r2_symmde_", t, collapse = "")] = templm$adj.r.squared

}#For X1 only

##using variables to interpret MDE or DYS

templm = summary(lm(as.formula(paste0("mdecause~",resu$variable[r], collapse = "")),sim))

resu[r, paste0("rereg_mde_", t, collapse = "")] = templm$coefficients[as.character(resu$variable[r]), "Estimate"]

resu[r, paste0("rereg_p_mde_", t, collapse = "")] = templm$coefficients[as.character(resu$variable[r]), "Pr(>|t|)"]

resu[r, paste0("rereg_int_mde_", t, collapse = "")] = templm$coefficients["(Intercept)", "Estimate"]

resu[r, paste0("rereg_intp_mde_", t, collapse = "")] = templm$coefficients["(Intercept)", "Pr(>|t|)"]

resu[r, paste0("rereg_r2_mde_", t, collapse = "")] = templm$adj.r.squared

#interpretation for the other related disease

templm = summary(lm(as.formula(paste0("dyscause~", resu$variable[r], collapse = "")),sim))

resu[r, paste0("rereg_dys_", t, collapse = "")] = templm$coefficients[as.character(resu$variable[r]), "Estimate"]

resu[r, paste0("rereg_p_dys_", t, collapse = "")] = templm$coefficients[as.character(resu$variable[r]), "Pr(>|t|)"]

resu[r, paste0("rereg_int_dys_", t, collapse = "")] = templm$coefficients["(Intercept)", "Estimate"]

resu[r, paste0("rereg_intp_dys_", t, collapse = "")] = templm$coefficients["(Intercept)", "Pr(>|t|)"]

resu[r, paste0("rereg_r2_dys_", t, collapse = "")] = templm$adj.r.squared

##Logit regression

##using variables to interpret MDE or DYS

templm = summary(glm(as.formula(paste0("mdecause~", paste0("X", 1:as.numeric(gsub("X", "", as.character(resu$variable[r]))), collapse = "+"), collapse = "")),sim, family = "binomial"))

resu[r, paste0("relog_mde_", t, collapse = "")] = paste0(templm$coefficients[paste("X", 1:as.numeric(gsub("X", "", as.character(resu$variable[r]))), sep = ""), "Estimate"], collapse = ",")

resu[r, paste0("relog_p_mde_", t, collapse = "")] = paste0(templm$coefficients[paste("X", 1:as.numeric(gsub("X", "", as.character(resu$variable[r]))), sep = ""), "Pr(>|z|)"], collapse = ",")

resu[r, paste0("relog_int_mde_", t, collapse = "")] = templm$coefficients["(Intercept)", "Estimate"]

resu[r, paste0("relog_intp_mde_", t, collapse = "")] = templm$coefficients["(Intercept)", "Pr(>|z|)"]

resu[r, paste0("relog_aic_mde_", t, collapse = "")] = templm$aic

resu[r, paste0("relog_deviance_mde_", t, collapse = "")] = templm$deviance

#interpretation for the other related disease

templm = summary(glm(as.formula(paste0("dyscause~", paste0("X", 1:as.numeric(gsub("X", "", as.character(resu$variable[r]))), collapse = "+"), collapse = "")),sim, family = "binomial"))

resu[r, paste0("relog_dys_", t, collapse = "")] = paste0(templm$coefficients[paste("X", 1:as.numeric(gsub("X", "", as.character(resu$variable[r]))), sep = ""), "Estimate"], collapse = ",")

resu[r, paste0("relog_p_dys_", t, collapse = "")] = paste0(templm$coefficients[paste("X", 1:as.numeric(gsub("X", "", as.character(resu$variable[r]))), sep = ""), "Pr(>|z|)"], collapse = ",")

resu[r, paste0("relog_int_dys_", t, collapse = "")] = templm$coefficients["(Intercept)", "Estimate"]

resu[r, paste0("relog_intp_dys_", t, collapse = "")] = templm$coefficients["(Intercept)", "Pr(>|z|)"]

resu[r, paste0("relog_aic_dys_", t, collapse = "")] = templm$aic

resu[r, paste0("relog_deviance_dys_", t, collapse = "")] = templm$deviance

#interpretation for the other symptoms

if(resu$variable[r] != "X1"){

templm = summary(glm(as.formula(paste0("X1~", paste0("X", 1:as.numeric(gsub("X", "", as.character(resu$variable[r]))), collapse = "+"), collapse = "")),sim, family = "binomial"))

resu[r, paste0("relog_sym_", t, collapse = "")] = paste0(templm$coefficients[paste("X", 2:as.numeric(gsub("X", "", as.character(resu$variable[r]))), sep = ""), "Estimate"], collapse = ",")

resu[r, paste0("relogse_sym_", t, collapse = "")] = paste0(templm$coefficients[paste("X", 2:as.numeric(gsub("X", "", as.character(resu$variable[r]))), sep = ""), "Std. Error"], collapse = ",")

resu[r, paste0("relog_p_sym_", t, collapse = "")] = paste0(templm$coefficients[paste("X", 2:as.numeric(gsub("X", "", as.character(resu$variable[r]))), sep = ""), "Pr(>|z|)"], collapse = ",")

resu[r, paste0("relog_int_sym_", t, collapse = "")] = templm$coefficients["(Intercept)", "Estimate"]

resu[r, paste0("relog_intp_sym_", t, collapse = "")] = templm$coefficients["(Intercept)", "Pr(>|z|)"]

resu[r, paste0("relog_aic_sym_", t, collapse = "")] = templm$aic

resu[r, paste0("relog_deviance_sym_", t, collapse = "")] = templm$deviance

}

# }#if variable %in% paste("X", 1:100, sep = "")

}#if variable %in% X1, X2...

}#variables not missing

##regression for the diagnosis

if(is.na(resu$variable[r]) == FALSE ){

#diagnositic accuracy

# library(pROC)

# library(caret)

if(length(unique(sim[, as.character(resu$variable[r])]))<= 2){

# there are issues with sim$X1 and neede

resu[r, paste0("sen_mde_", t, collapse = "")] = nrow(sim[which(sim$mdecause == 1 & sim[,as.character(resu$variable[r])] == 1),])/nrow(sim[which(sim$mdecause == 1),])

resu[r, paste0("spe_mde_", t, collapse = "")] = nrow(sim[which(sim$mdecause == 0 & sim[,as.character(resu$variable[r])] == 0),])/nrow(sim[which(sim$mdecause == 0),])

resu[r, paste0("ppv_mde_", t, collapse = "")] = nrow(sim[which(sim$mdecause == 1 & sim[,as.character(resu$variable[r])] == 1),])/nrow(sim[which(sim[,as.character(resu$variable[r])] == 1),])

resu[r, paste0("npv_mde_", t, collapse = "")] = nrow(sim[which(sim$mdecause == 0 & sim[,as.character(resu$variable[r])] == 0),])/nrow(sim[which(sim[,as.character(resu$variable[r])] == 0),])

resu[r, paste0("sen_dys_", t, collapse = "")] = nrow(sim[which(sim$dyscause == 1 & sim[,as.character(resu$variable[r])] == 1),])/nrow(sim[which(sim$dyscause == 1),])

resu[r, paste0("spe_dys_", t, collpase = "")] = nrow(sim[which(sim$dyscause == 0 & sim[,as.character(resu$variable[r])] == 0),])/nrow(sim[which(sim$dyscause == 0),])

resu[r, paste0("ppv_dys_", t, collpase = "")] = nrow(sim[which(sim$dyscause == 1 & sim[,as.character(resu$variable[r])] == 1),])/nrow(sim[which(sim[,as.character(resu$variable[r])] == 1),])

resu[r, paste0("npv_dys_", t, collpase = "")] = nrow(sim[which(sim$dyscause == 0 & sim[,as.character(resu$variable[r])] == 0),])/nrow(sim[which(sim[,as.character(resu$variable[r])] == 0),])

resu[r, paste0("sen_man_", t, collapse = "")] = nrow(sim[which(sim$maniccause == 1 & sim[,as.character(resu$variable[r])] == 1),])/nrow(sim[which(sim$maniccause == 1),])

resu[r, paste0("spe_man_", t, collapse = "")] = nrow(sim[which(sim$maniccause == 0 & sim[,as.character(resu$variable[r])] == 0),])/nrow(sim[which(sim$maniccause == 0),])

resu[r, paste0("ppv_man_", t, collapse = "")] = nrow(sim[which(sim$maniccause == 1 & sim[,as.character(resu$variable[r])] == 1),])/nrow(sim[which(sim[,as.character(resu$variable[r])] == 1),])

resu[r, paste0("npv_man_", t, collapse = "")] = nrow(sim[which(sim$maniccause == 0 & sim[,as.character(resu$variable[r])] == 0),])/nrow(sim[which(sim[,as.character(resu$variable[r])] == 0),])

#AUC not for each variable, for the sum of all X variables

if(as.character(resu$variable[r]) %in% paste("X", 2:nvar, sep = "")){

pROC_obj <- roc(sim$mdecause, rowSums(sim[, paste("X", 1:as.numeric(gsub("X", "", as.character(resu$variable[r]))), sep = "")]) ,

levels = c(0,1),

direction=c("<"),

smoothed = TRUE,

# arguments for ci

ci=TRUE, ci.alpha=0.95, stratified=FALSE,

# arguments for plot

plot=F)

resu[r, paste0("auc_mde_", t, collapse = "")] = pROC_obj$auc

resu[r, paste0("auc_mde_n_", t, collapse = "")] = pROC_obj$thresholds[which.max(abs(pROC_obj$sensitivities+pROC_obj$specificities-1))]

resu[r, paste0("auc_mde_95lo_", t, collapse = "")] = pROC_obj$ci[1]

resu[r, paste0("auc_mde_95up_", t, collapse = "")] = pROC_obj$ci[3]

resu[r, paste0("sen_mde_max_", t, collapse = "")] = pROC_obj$sensitivities[which.max(abs(pROC_obj$sensitivities+pROC_obj$specificities-1))]

resu[r, paste0("spe_mde_max_", t, collapse = "")] = pROC_obj$specificities[which.max(abs(pROC_obj$sensitivities+pROC_obj$specificities-1))]

resu[r, paste0("sen_mde_maxn_", t, collapse = "")] = pROC_obj$thresholds[which.max(abs(pROC_obj$sensitivities+pROC_obj$specificities-1))]

resu[r, paste0("spe_mde_maxn_", t, collapse = "")] = pROC_obj$thresholds[which.max(abs(pROC_obj$sensitivities+pROC_obj$specificities-1))]

}##If not X1

if(as.character(resu$variable[r]) %in% paste("X", 1, sep = "")){

pROC_obj <- roc(sim$mdecause, sim[, as.character(resu$variable[r])],

levels = c(0,1),

direction=c("<"),

smoothed = TRUE,

# arguments for ci

ci=TRUE, ci.alpha=0.95, stratified=FALSE,

# arguments for plot

plot=F)

resu[r, paste0("auc_mde_", t, collapse = "")] = pROC_obj$auc

resu[r, paste0("auc_mde_n_", t, collapse = "")] = pROC_obj$thresholds[which.max(abs(pROC_obj$sensitivities+pROC_obj$specificities-1))]

resu[r, paste0("auc_mde_95lo_", t, collapse = "")] = pROC_obj$ci[1]

resu[r, paste0("auc_mde_95up_", t, collapse = "")] = pROC_obj$ci[3]

resu[r, paste0("sen_mde_max_", t, collapse = "")] = pROC_obj$sensitivities[which.max(abs(pROC_obj$sensitivities+pROC_obj$specificities-1))]

resu[r, paste0("spe_mde_max_", t, collapse = "")] = pROC_obj$specificities[which.max(abs(pROC_obj$sensitivities+pROC_obj$specificities-1))]

}

#manic as outcome for diagnostic test accuracy

if(resu$variable[r] %in% paste("X", 2:nvar, sep = "")){

# sim$sum =

pROC_obj <- roc(sim$maniccause, rowSums(sim[, paste("X", 1:as.numeric(gsub("X", "", resu$variable[r])), sep = "")]),

levels = c(0,1),

direction=c("<"),

smoothed = TRUE,

# arguments for ci

ci=TRUE, ci.alpha=0.95, stratified=FALSE,

# arguments for plot

plot=F)

resu[r, paste0("auc_man_", t, collapse = "")] = pROC_obj$auc

resu[r, paste0("auc_man_n_", t, collapse = "")] = pROC_obj$thresholds[which.max(abs(pROC_obj$sensitivities+pROC_obj$specificities-1))]

resu[r, paste0("auc_man_95lo_", t, collapse = "")] = pROC_obj$ci[1]

resu[r, paste0("auc_man_95up_", t, collapse = "")] = pROC_obj$ci[2]

resu[r, paste0("sen_man_max_", t, collapse = "")] = pROC_obj$sensitivities[which.max(abs(pROC_obj$sensitivities+pROC_obj$specificities-1))]

resu[r, paste0("spe_man_max_", t, collapse = "")] = pROC_obj$specificities[which.max(abs(pROC_obj$sensitivities+pROC_obj$specificities-1))]

}

if(resu$variable[r] %in% paste("X", 1, sep = "")){

pROC_obj <- roc(sim$maniccause, sim[, as.character(resu$variable[r])],

levels = c(0,1),

direction=c("<"),

smoothed = TRUE,

# arguments for ci

ci=TRUE, ci.alpha=0.95, stratified=FALSE,

# arguments for plot

plot=F)

resu[r, paste0("auc_man_", t, collapse = "")] = pROC_obj$auc

resu[r, paste0("auc_man_n_", t, collapse = "")] = 1

resu[r, paste0("auc_man_95lo_", t, collapse = "")] = pROC_obj$ci[1]

resu[r, paste0("auc_man_95up_", t, collapse = "")] = pROC_obj$ci[2]

resu[r, paste0("sen_man_max_", t, collapse = "")] = pROC_obj$sensitivities[2]

resu[r, paste0("spe_man_max_", t, collapse = "")] = pROC_obj$specificities[2]

}

#dys as outcome for diagnostic test accuracy

if(resu$variable[r] %in% paste("X", 2:nvar, sep = "")){

# sim$sum =

pROC_obj <- roc(sim$dyscause, rowSums(sim[, paste("X", 1:as.numeric(gsub("X", "", resu$variable[r])), sep = "")]),

levels = c(0,1),

direction=c("<"),

smoothed = TRUE,

# arguments for ci

ci=TRUE, ci.alpha=0.95, stratified=FALSE,

# arguments for plot

plot=F)

resu[r, paste0("auc_dys_", t, collapse = "")] = pROC_obj$auc

resu[r, paste0("auc_dys_n_", t, collapse = "")] = pROC_obj$thresholds[which.max(abs(pROC_obj$sensitivities+pROC_obj$specificities-1))]

resu[r, paste0("auc_dys_95lo_", t, collapse = "")] = pROC_obj$ci[1]

resu[r, paste0("auc_dys_95up_", t, collapse = "")] = pROC_obj$ci[2]

resu[r, paste0("sen_dys_max_", t, collapse = "")] = pROC_obj$sensitivities[which.max(abs(pROC_obj$sensitivities+pROC_obj$specificities-1))]

resu[r, paste0("spe_dys_max_", t, collapse = "")] = pROC_obj$specificities[which.max(abs(pROC_obj$sensitivities+pROC_obj$specificities-1))]

}

if(resu$variable[r] %in% paste("X", 1, sep = "")){

pROC_obj <- roc(sim$dyscause, sim[, as.character(resu$variable[r])],

levels = c(0,1),

direction=c("<"),

smoothed = TRUE,

# arguments for ci

ci=TRUE, ci.alpha=0.95, stratified=FALSE,

# arguments for plot

plot=F)

resu[r, paste0("auc_dys_", t, collapse = "")] = pROC_obj$auc

resu[r, paste0("auc_dys_n_", t, collapse = "")] = pROC_obj$thresholds[which.max(abs(pROC_obj$sensitivities+pROC_obj$specificities-1))]

resu[r, paste0("auc_dys_95lo_", t, collapse = "")] = pROC_obj$ci[1]

resu[r, paste0("auc_dys_95up_", t, collapse = "")] = pROC_obj$ci[2]

resu[r, paste0("sen_dys_max_", t, collapse = "")] = pROC_obj$sensitivities[2]

resu[r, paste0("spe_dys_max_", t, collapse = "")] = pROC_obj$specificities[2]

}

}#if more than or equal to 2 levels

}#if resu$variable[r] not NA

if(r %in% as.character(1:200*10)){print(c("r:", r))}

}#r = rows of the variable list

print(c("At risk:", dxrisk[dr]))

print(c("Dx cor:", dxcor[dc]))

print(c("Sym cor:", symcor[sc]))

print(c("Relative risk:", relativer[rr]))

print(c("Dx Prevalence: ", dxpreval[dp]))

print(c("Times:", t))

print(Sys.time())

}#times

write.csv(resu, file = paste0("simulation results_dxcor", dxcor[dc], "_dxrisk", dxrisk[dr], "_rr", relativer[rr], "_dxpreval", dxpreval[dp], "_symcor", symcor[sc], "_symcordi", symcordi[scd], ".csv", collapse = ""))

print(c("At risk:", dxrisk[dr]))

print(c("Dx cor:", dxcor[dc]))

print(c("Sym cor:", symcor[sc]))

print(c("At risk:", dxrisk[dr]))

print(c("RR:", relativer[rr]))

print(c("Dx Prevalence: ", dxpreval[dp]))

print(Sys.time())

# print(c("Assumption: ", assum[ass]))

}#to keep the rmbin running ,if(symcor[sc]*(as.numeric(relativer[rr]) * as.numeric(dxpreval[dp])) > 0.3)

}#sym cor di

}#symcor: symptom correlations

}#dxpreval: disease incidence

}#relative risks

gc()

}#proportions of populations at risk

}#true disease correlations

```

# Appendix 2

Figure 1. Assumed and derived baseline symptom incidence among those not diseased


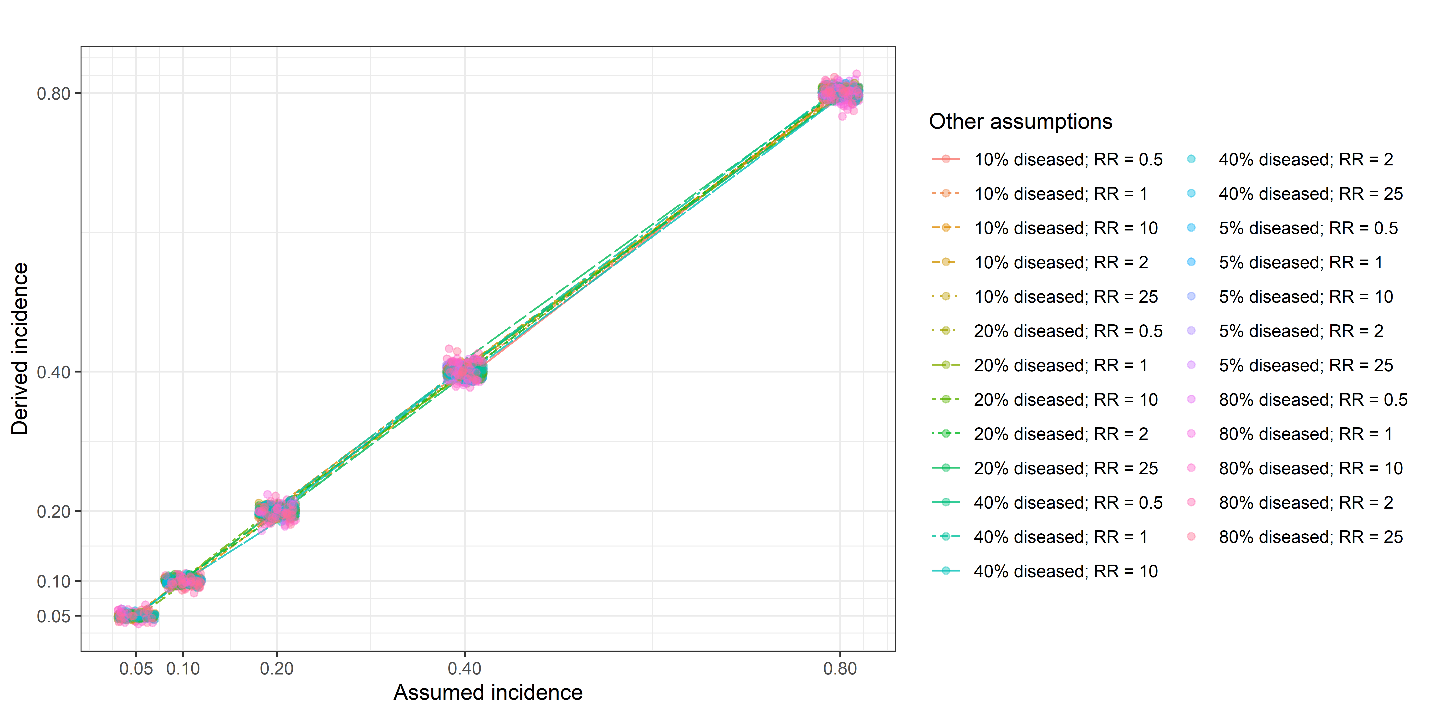


RR = risk ratio

Figure 2. Assumed and derived risk ratios


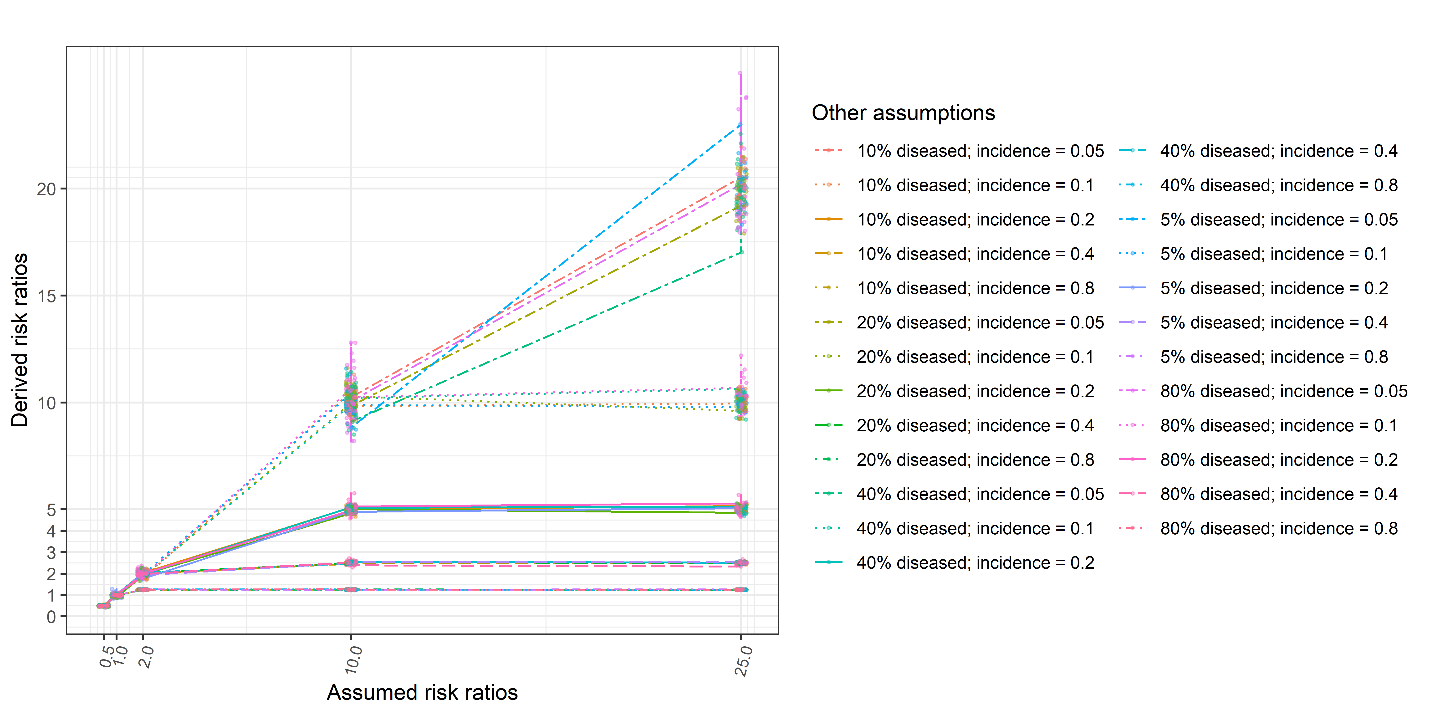


# Appendix 3

Figure 3: risk ratio as 2, baseline incidence as 0.1, proportions diseased as 0.05, no correlations between diseases, and no correlations between symptoms


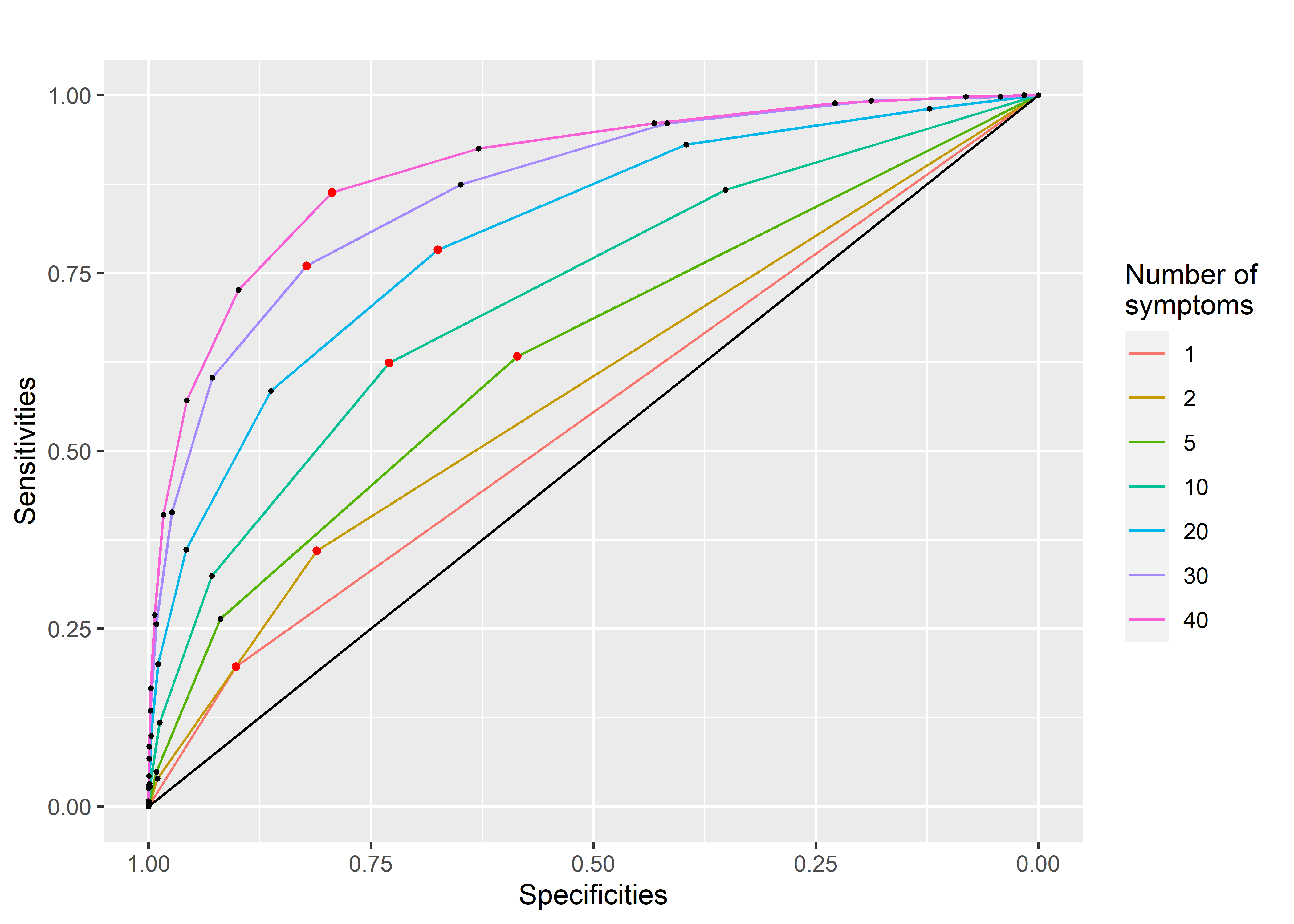


Red dots = the set of sensitivities and specificities with the largest difference in the absolute values between 1 and the sums of sensitivities and specificities. For each number of symptoms used for disease prediction, one red dot, best set of sensitivities and specificities, was selected.

Figure 4: risk ratio as 0.5, baseline incidence as 0.4, proportions diseased as 0.2, no correlations between diseases, and 0.8 correlations between symptoms


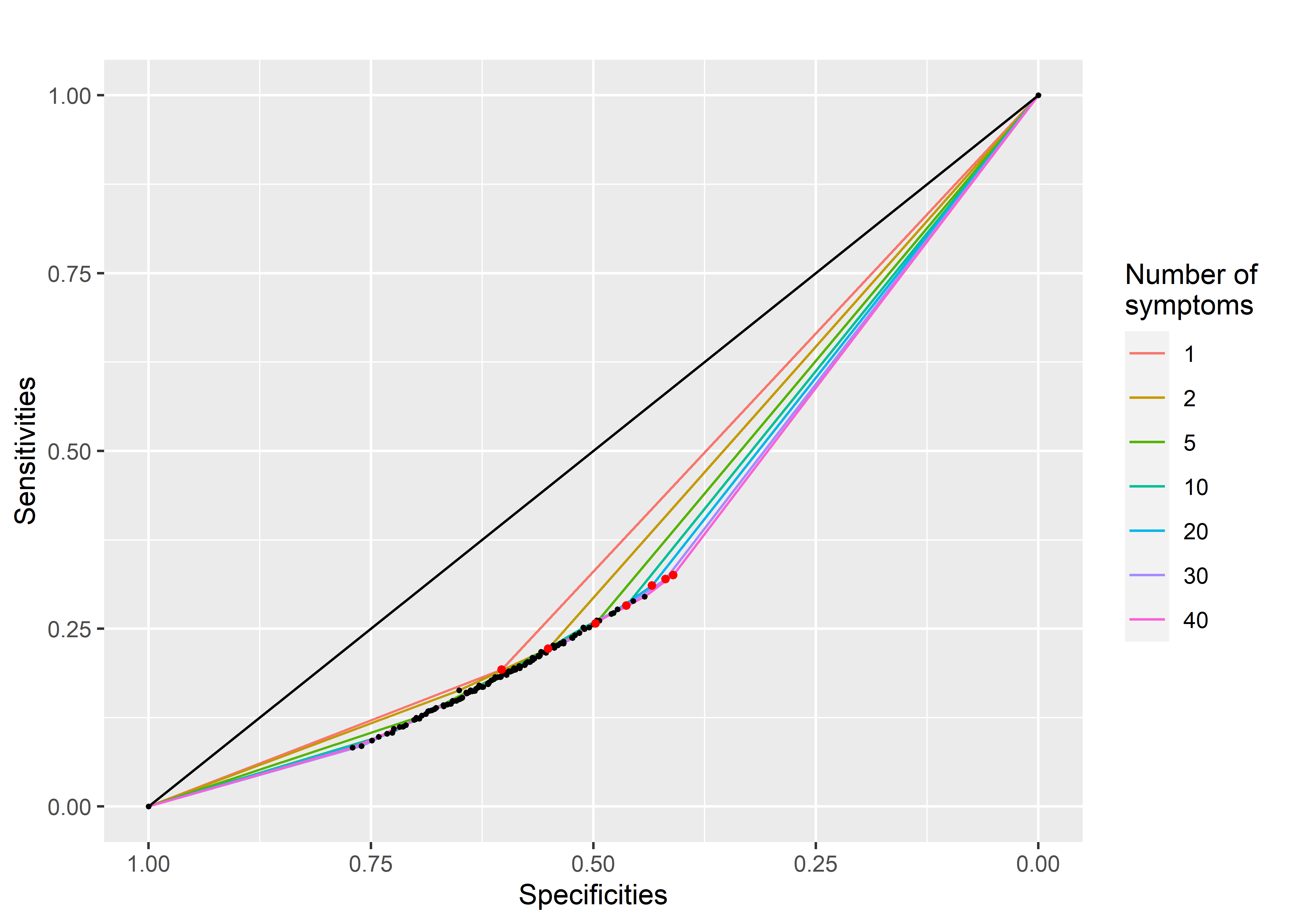


Red dots = the set of sensitivities and specificities with the largest difference in the absolute values between 1 and the sums of sensitivities and specificities. For each number of symptoms used for disease prediction, one red dot, best set of sensitivities and specificities, was selected.

Figure 5: risk ratio as 0.5, baseline incidence as 0.8, proportions diseased as 0.2, no correlations between diseases, and 0.8 correlations between symptoms


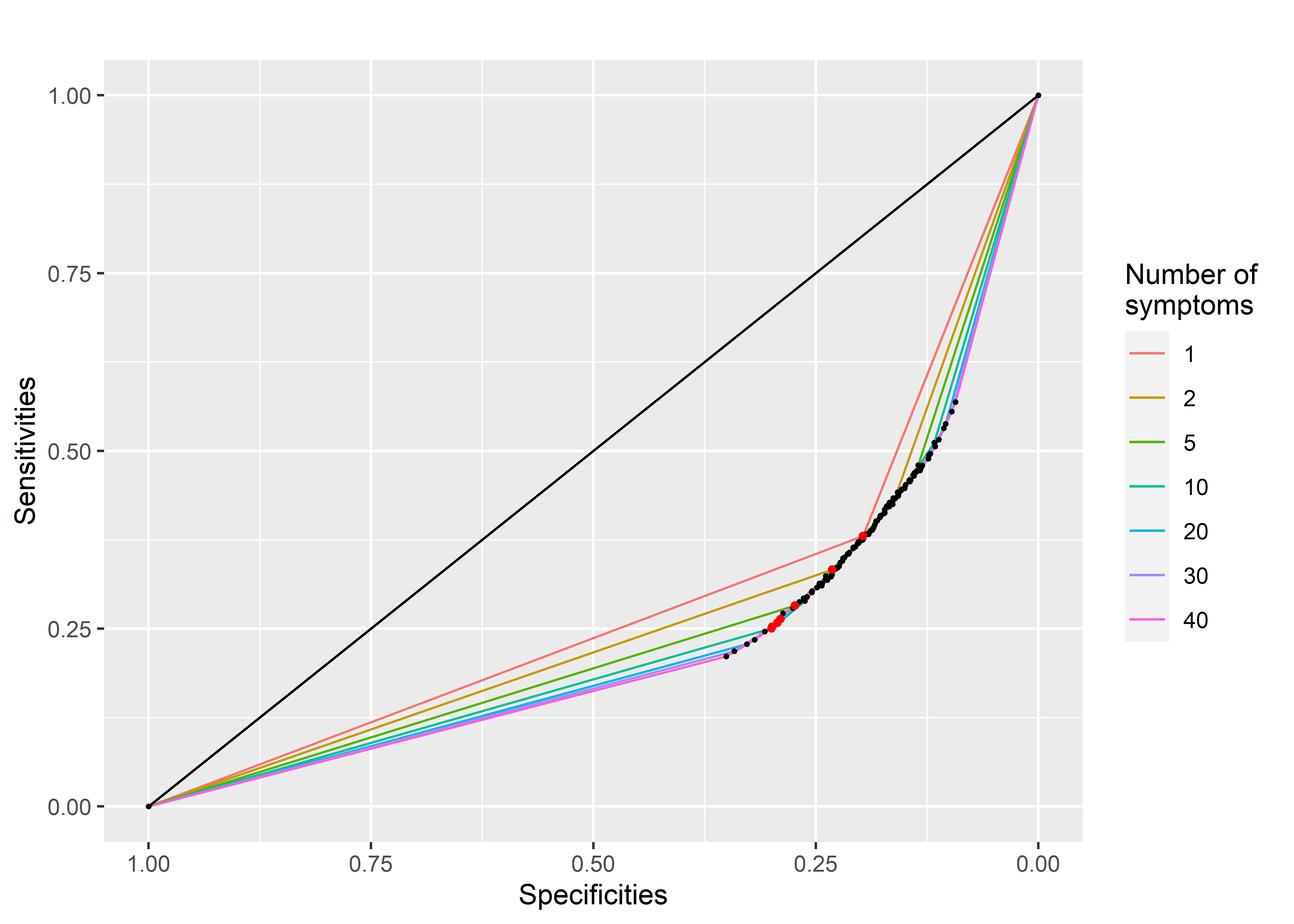


Red dots = the set of sensitivities and specificities with the largest difference in the absolute values between 1 and the sums of sensitivities and specificities. For each number of symptoms used for disease prediction, one red dot, best set of sensitivities and specificities, was selected.

Figure 6: risk ratio as 2, baseline incidence as 0.1, proportions diseased as 0.05, no correlations between diseases, and no correlations between symptoms


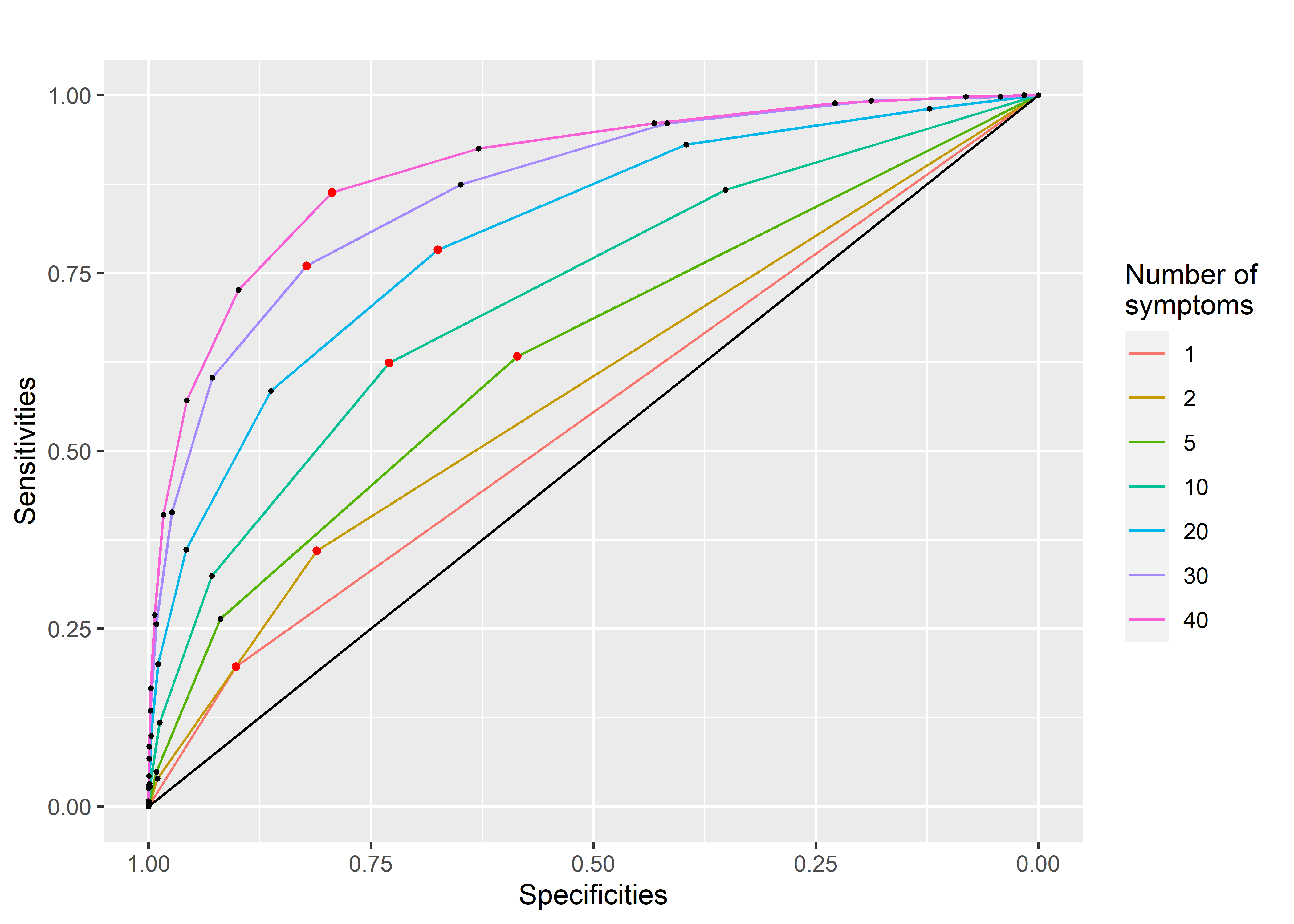


Red dots = the set of sensitivities and specificities with the largest difference in the absolute values between 1 and the sums of sensitivities and specificities. For each number of symptoms used for disease prediction, one red dot, best set of sensitivities and specificities, was selected.

Figure 7: risk ratio as 2, baseline incidence as 0.1, proportions diseased as 0.05, no correlations between diseases, and no correlations between symptoms


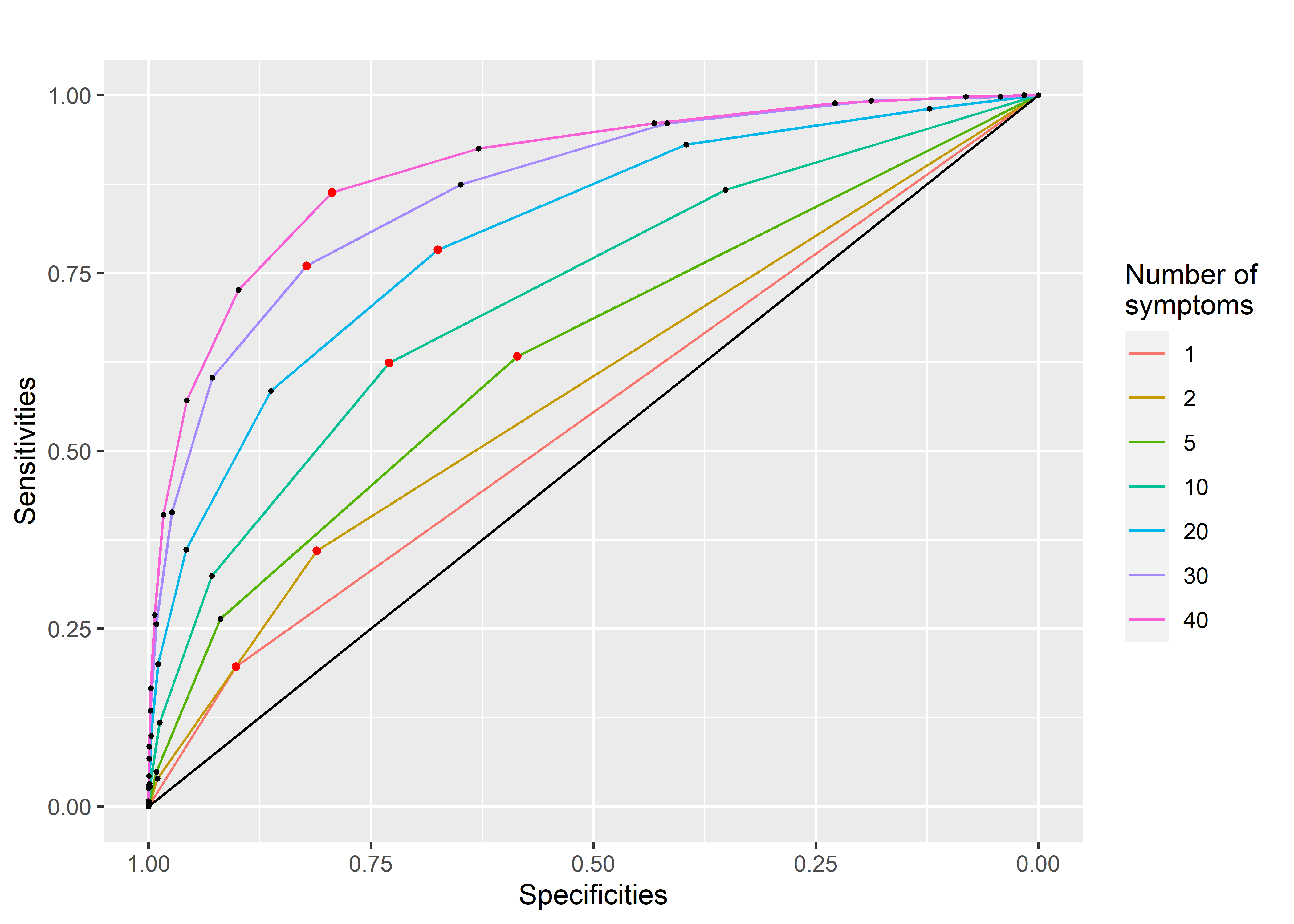


Red dots = the set of sensitivities and specificities with the largest difference in the absolute values between 1 and the sums of sensitivities and specificities. For each number of symptoms used for disease prediction, one red dot, best set of sensitivities and specificities, was selected.

Figure 8: risk ratio as 2, baseline incidence as 0.4, proportions diseased as 0.05, no correlations between diseases, and 0.8 correlations between symptoms


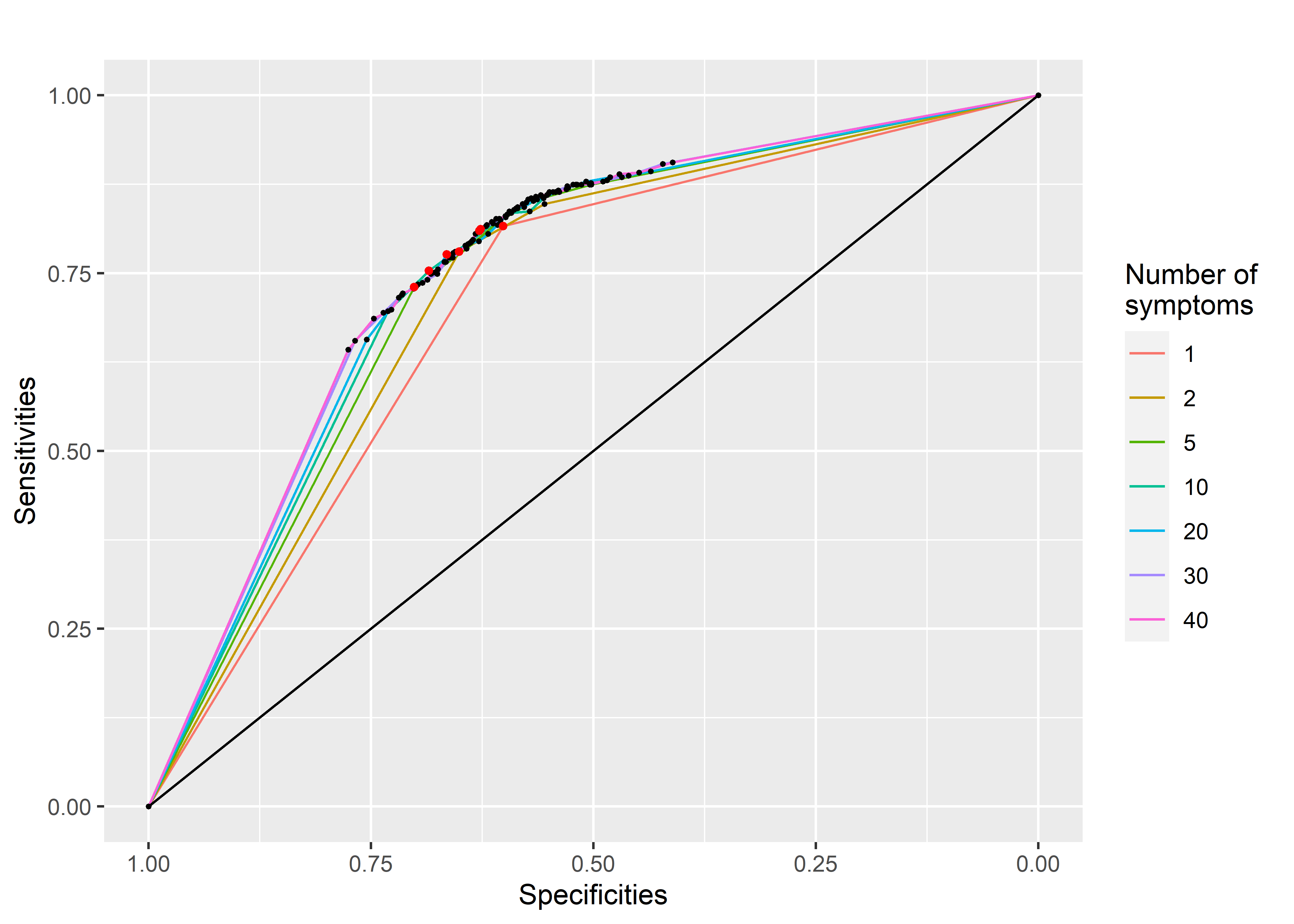


Red dots = the set of sensitivities and specificities with the largest difference in the absolute values between 1 and the sums of sensitivities and specificities. For each number of symptoms used for disease prediction, one red dot, best set of sensitivities and specificities, was selected.

Figure 9: risk ratio as 25, baseline incidence as 0.2, proportions diseased as 0.1, 0.7 correlations between diseases, and 0.8 correlations between symptoms


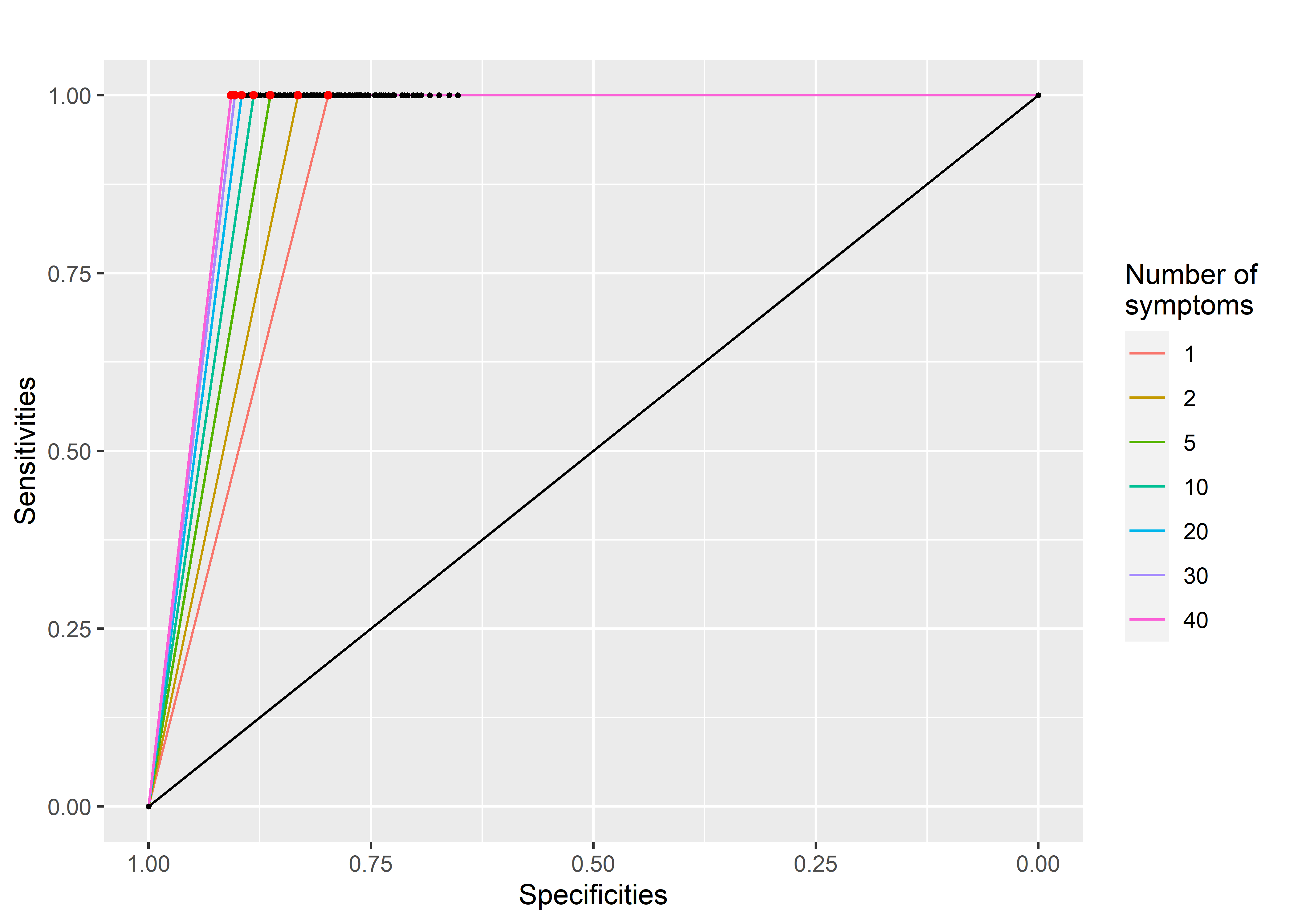


Red dots = the set of sensitivities and specificities with the largest difference in the absolute values between 1 and the sums of sensitivities and specificities. For each number of symptoms used for disease prediction, one red dot, best set of sensitivities and specificities, was selected.

# Appendix 4

Figure 10. The area under the receiver operating characteristic curve based on numbers of symptoms, baseline symptom incidence, and symptom risk ratios, symptom correlations among those not diseased as 0.8.


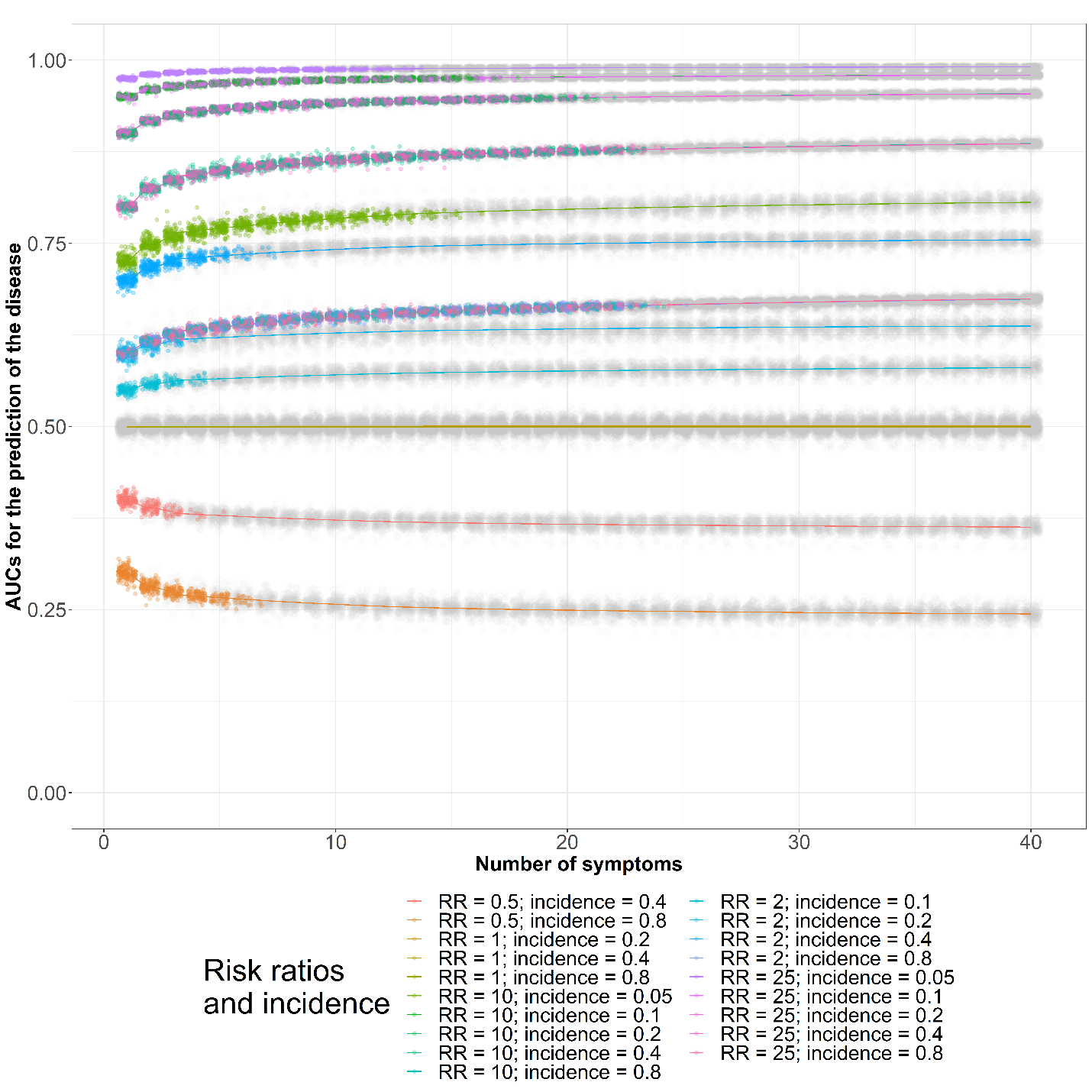


RR = risk ratio; incidence = baseline symptom incidence among those not diseased.

Gray dots were the area under curve (AUCs) whose 95% confidence intervals overlapped with those of the maximal AUCs identified using a maximum of 40 symptoms for disease prediction.

All AUCs assuming 0.8 correlations between symptoms among those not diseased were illustrated.

Figure 11. The area under the receiver operating characteristic curve based on numbers of symptoms, baseline symptom incidence, and symptom risk ratios, symptom correlations among those not diseased as 0.4.


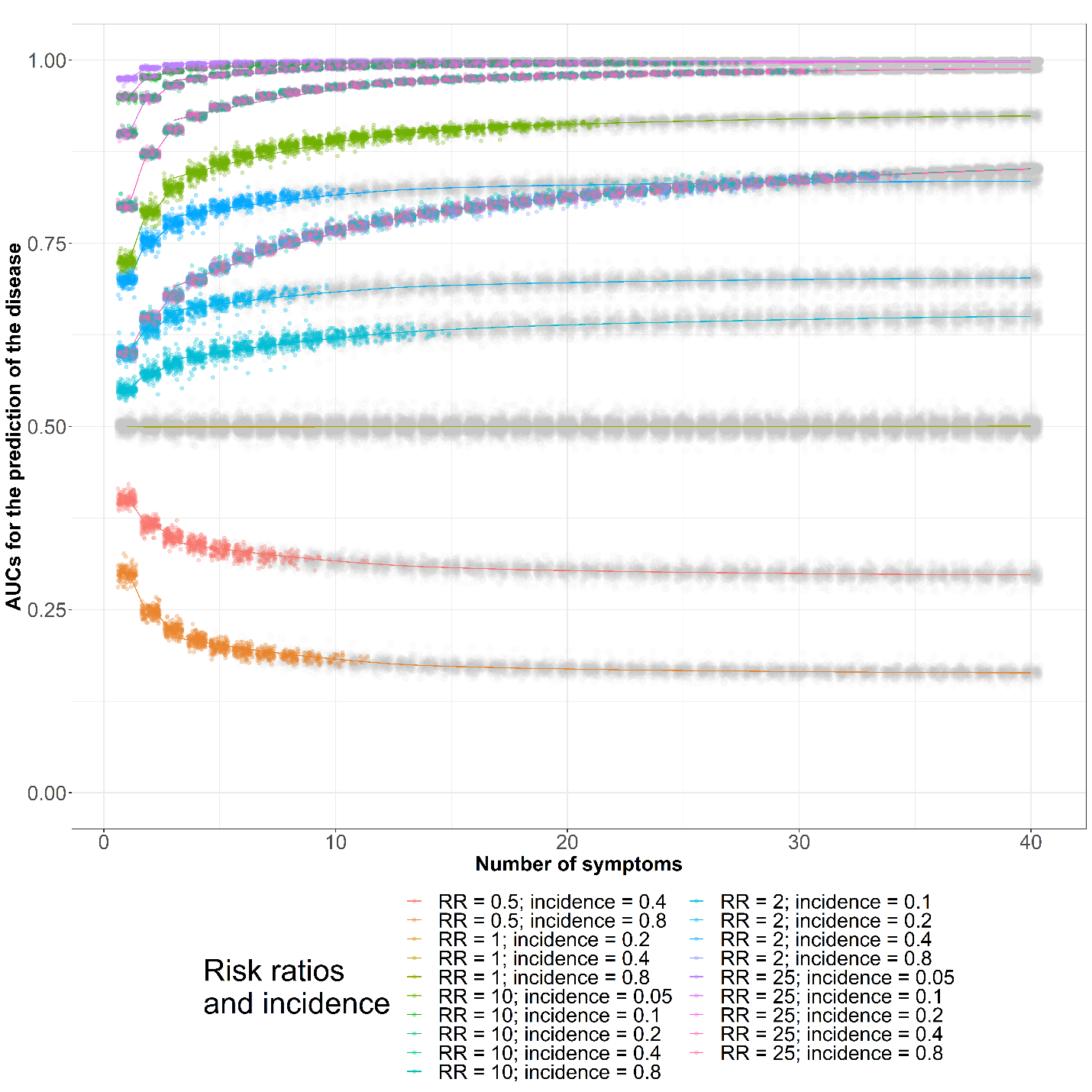


RR = risk ratio; incidence = baseline symptom incidence among those not diseased.

Gray dots were the area under curve (AUCs) whose 95% confidence intervals overlapped those of the maximal AUCs identified using a maximum of 40 symptoms for disease prediction.

Figure 12. The area under the receiver operating characteristic curve based on numbers of symptoms, baseline symptom incidence, and risk ratios, symptom correlations among those not diseased as 0.


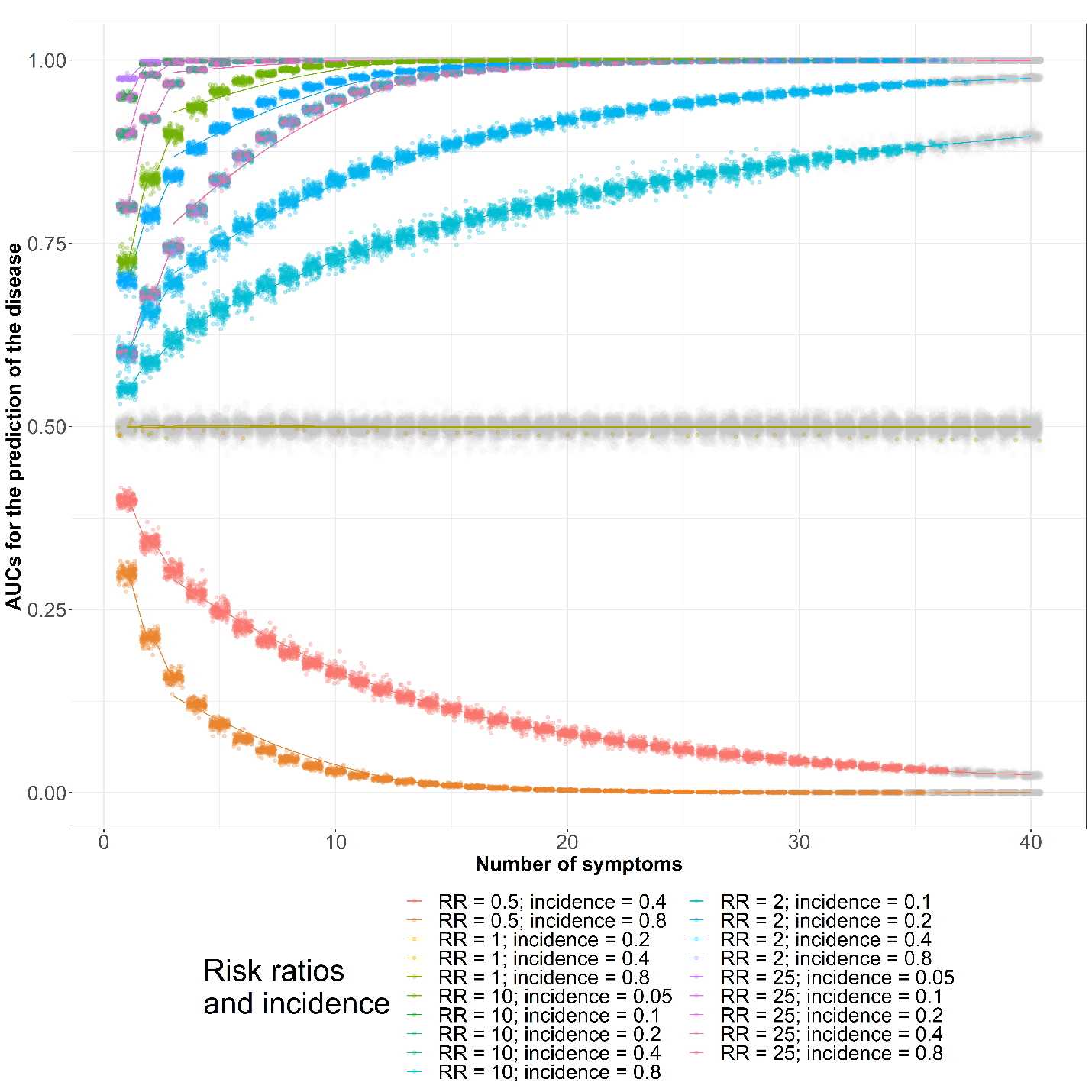


RR = risk ratio; incidence = baseline symptom incidence among those not diseased.

Gray dots were the area under curve (AUCs) whose 95% confidence intervals overlapped those of the maximal AUCs identified using a maximum of 40 symptoms for disease prediction.
